# Adaptation in structured populations and fuzzy boundaries between hard and soft sweeps

**DOI:** 10.1371/journal.pcbi.1007426

**Published:** 2019-11-11

**Authors:** Yichen Zheng, Thomas Wiehe

**Affiliations:** Institute for Genetics, University of Cologne, Cologne, Germany; Uppsala Universitet, SWEDEN

## Abstract

Selective sweeps, the genetic footprint of positive selection, have been extensively studied in the past decades, with dozens of methods developed to identify swept regions. However, these methods suffer from both false positive and false negative reports, and the candidates identified with different methods are often inconsistent with each other. We propose that a biological cause of this problem can be population subdivision, and a technical cause can be incomplete, or inaccurate, modeling of the dynamic process associated with sweeps. Here we used simulations to show how these effects interact and potentially cause bias. In particular, we show that sweeps maybe misclassified as either hard or soft, when the true time stage of a sweep and that implied, or pre-supposed, by the model do not match. We call this “temporal misclassification”. Similarly, “spatial misclassification (softening)” can occur when hard sweeps, which are imported by migration into a new subpopulation, are falsely identified as soft. This can easily happen in case of local adaptation, i.e. when the sweeping allele is not under positive selection in the new subpopulation, and the underlying model assumes panmixis instead of substructure. The claim that most sweeps in the evolutionary history of humans were soft, may have to be reconsidered in the light of these findings.

## Author summary

Identifying the traces of adaptive evolution is still difficult, in particular when populations are not in equilibrium. Using forward-in-time simulations, we studied adaptation by selective sweeps in populations that are divided into demes with limited migration among them. We applied different sweep tests, whose sensitivities are found to vary widely across demographic scenarios and temporal stages. First, the temporal stage of a sweep (ongoing vs completed) significantly affects detection, especially when machine learning algorithms are used and training and test stages do not match. Second, imported alleles from a neighboring deme with local adaptation can result in spurious sweep signals. In both cases, signals are often detected as “soft sweeps” (adaptation from standing variation) while in fact they are “hard sweeps” (adaptation from single mutation), originating in the same subpopulation in the former case and in some other subpopulation in the latter case. We call these phenomena “temporal” and “spatial softening”. Finally, under low migration, the time window in which a sweep can be detected becomes very narrow, and power tends to be low. Generally, however, haplotype-based methods seem to be less affected than frequency-spectrum-based tests.

## Introduction

### Methods to detect selective sweeps from population sequence data

Selective sweeps were originally studied in the context of panmictic populations of constant size [1], [2], [3], [4], and later also in scenarios of changing population size or population substructure (e.g., [5], [6], [7]). A large array of statistical methods is available, and is routinely applied in genome annotation studies, to identify genomic locations which supposedly have experienced recent selective sweeps (reviewed by [8]). These methods can roughly be grouped into three types, or mixtures of them, according to their underlying theoretical considerations: there are statistical tests for sweeps which are based on the site frequency spectrum (SFS) (see [9]), tests which are based on haplotypes and their distribution [10], [11], and tests based on properties of the inferred genealogical tree of a sequence sample [12], [13]. Some of the well established methods such as the likelihood ratio test (LRT) of e.g., [14], [15], linkage-disequilibrium (LD) [16] and Bayesian or Hidden-Markov-Model- (HMM-)based methods [17], [18], [19] can be considered as combinations of these types.

More recently, machine learning methods have been used to detect selective sweeps [20]. Such algorithms have no pre-specified concrete model or formulas, but rather use training datasets (*train-sets*) with known classification (selection or not) to create predictors. The predictor that separates a number of data points (loci) into categories is called a *classifier*. This has the advantage of (1) combining the power of a multitude of summary statistics, and (2) being unaffected by any *a priori* assumptions regarding *how* sweeps can affect the output. The downside of machine learning is that (1) the predictor algorithm is usually not easily human-interpretable, in the sense that it is difficult, or impossible, to understand the algorithmic basis of the classification. Furthermore, train- and test-sets must have the same, or at least similar, demographic parameters so that demographic effects will not be mis-identified as selection signals. While robustness with respect to demographic effects is often tested in sweep-detection methods (e.g., [15], [21]), such tests often limited to population size changes, and real population histories may lie outside of the tested parameter space.

### Selective sweeps in models of population subdivision

Demographic assumptions for standard population genetic models, such as panmictic and constant-sized populations, are usually unrealistic for biological data. Demographic effects may mimic or complicate the genetic footprint of selective sweeps [22]. A common complication is population subdivision, where multiple sub-populations (demes) exchange a limited amount of migrants per generation, leading to partial differentiation and a neutral frequency spectrum that is different from that of panmictic populations. We refer to the deme where the adaptive mutation arises as the native deme, and the deme where the adaptive mutation is imported to as the non-native deme. The number of migrants per generation, *Nm* (i.e. the effective population size *N* multiplied by migration rate *m* per individual per generation), can vary across multiple orders of magnitude, resulting in a wide spectrum of different scenarios. In addition, an adaptive allele which causes a selective sweep can be globally adaptive (i.e., beneficial in all demes), locally adaptive (i.e., neutral in non-native demes or demes where it is imported to) or even negatively selected in non-native demes, leading to a large number of different cases.

In earlier studies of selective sweeps in subdivided populations, the impact of a globally adaptive allele sweeping through multiple demes by migration was analytically described [23], [24]. It was noted that the difference between original and imported haplotypes can *increase* inter-deme differentiation in linked loci despite global adaptation. Such linked loci can even experience an increase in genetic diversity if rare alleles only manage to hitchhike to an intermediate frequency [25]. Divergent selection (selection of different genotypes in different demes) increases differentiation resulting in a local maximum, because different haplotypes are fixed in different demes. In comparison, global adaptation results in two local differentiation maxima around the adaptive site instead of a single one [26]. This is because recombination on both sides causes different haplotypes to be associated with the same adaptive allele, and subsequently rise to high frequency in different demes [24]; this does not happen in the middle (immediate surrounding of adaptive allele) because there is not enough recombination. Another study explored the speed of an adaptation fixing in a sub-structured population [27], distinguishing three situations based on different migration rates. If the migration rate is high, the fixation will not be delayed, i.e. be as fast as in a panmictic population. If the migration rate is intermediate, non-native demes will slowly, but steadily, receive migrants with the adaptive allele; fixation slows down linearly with the logarithm of the migration rate. If the migration rate is so low that the first migrant occurs after fixation of the adaptation in the native deme where it originated from, the fixation time has an exponentially distributed component and is highly unpredictable. Another deterministic model examined how different migration rates affect homogenization of neutral loci linked to the adaptive allele across demes [28]. A lower migration rate increases the time delay between fixations of the adaptation in the demes, provides more time and chance for the beneficial and neutral loci to become unlinked, thus increasing the differentiation between the native and non-native demes.

Stochastic models, often based on simulations, attempt to determine how the randomness in migrating haplotypes and the sweeping process affect the genetic structure during and after sweeps. A simulation study [29] examined how sweeps in a subdivided population affect the frequency spectrum. It was found that population subdivision with low migration results in weaker depletion of intermediate-frequency alleles, but an enhanced effect of increasing linkage disequilibrium. It was also found that the sweep signals along the migration route are too erratic to be traced. On the other end of the parameter space, a recent study examined how several haplotype-based methods perform with local selection and high migration rates [30]. They found that XPCLR [15] performs well when a neighboring deme, without selection, can be used as a control group; however, none of the examined methods appears to be consistently powerful across different stages of the sweep.

### Hard and soft sweeps, and how to distinguish them

Selection from standing variation or recurrent adaptive mutations may lead to so-called “soft sweeps” [31], [32], [33]. One hallmark of soft sweeps is the presence of multiple haplotypes at the selected locus after its completion and a less pronounced reduction of nucleotide diversity than under a hard (classical) sweep. Therefore, soft sweeps are more difficult to detect (but see [33] and [21]), especially when using the mutation site frequency spectrum (SFS). Distinguishing hard from soft sweeps can be accomplished by combining SFS- and LD-statistics [33], [34]. If only one type of statistic is used, soft sweeps may only manifest as quantitatively weaker selective sweeps. To summarize the results from multiple statistics and to detect and classify sweeps is essentially a problem of dimension reduction, for which machine learning methods can be powerful tools. When implemented as supervised learning methods, pre-classified data (i.e. data for which the presence and type of sweeps are known) are needed to train and to test the model. Typically, these are obtained from simulations. After training and testing, experimental datasets with unknown selection status can be analyzed.

Assessing the relative roles of hard and soft sweeps has been a heatedly discussed matter of debate. For instance, it has been claimed that over 90% of the recent adaptation events in *Homo sapiens* have been soft sweeps, making hard sweeps the exception rather than the rule [35]. This finding is consistent with an earlier study reporting that “classic selective sweeps,” i.e., those characterized by a sharp reduction of diversity around an adaptive locus, are rare in human populations [36]. Also, artificial selection, which by definition involves a change of selective pressures, has been shown to mostly result in selection from standing variation [37], [38]. There are, however, arguments that soft sweeps have been over-emphasized [39]. In contrast, recent *Drosophila* adaptations are largely attributed to hard sweeps [40], [41] (but see [42]).

Aforementioned demographic processes can further complicate the classification of sweeps in at least two ways. First, demographic effects can alter the type of selective sweep: for instance, bottlenecks, together with drift, can cause all but one adapted haplotype to become lost, turning a soft sweep into an apparent hard sweep [22]. Second, demographic effects can affect summary statistics and cause mis-classification. In one of the original “soft sweep” papers, recurrent migration is mentioned as one cause of soft sweeps [32]. However, in their model the migration source population has the adaptive allele fixed “since a long time ago,” thus the migrating haplotypes are not more related to each other than expected under a neutral coalescent model. This may not be the case if the adaptive alleles fix in the demes in quick succession.

In summary, classification of selective sweeps under population substructure still entails a number of open problems. Here, we present the results of a simulation-based study to investigate and quantify the ability of different methods to detect sweeps in a variety of scenarios, including panmixis, population substructure, global and local adaptation, and different temporal stages of the sweep process. In particular, we examine the differentiation between hard and soft sweeps and the conditions when misclassification (especially as “hard” instead of “soft”) is likely. Future genomic studies, seeking to identify traces of adaptive selection in structured populations, should benefit from these results.

## Materials and methods

### Model and simulations

#### Allele frequency trajectory at a single site

The single-site simulations were conducted with a custom Perl script in a forward-in-time algorithm, considering migration, drift and selection. An adaptive allele appears in a previously homogeneous population initially in one individual in deme *d*_1_ and eventually fixes in both demes *d*_1_ and *d*_2_. We record the allele frequency in both demes every generation, and discard the run if the adaptive allele is lost. In addition, we simulated three-deme scenarios where the adaptive allele originates in a (hidden) deme *d*_0_ and is imported to *d*_1_ and *d*_2_ by migration. The selection coefficient can vary between the demes; in particular, for the three-deme model we considered situations where the focal allele is favored in *d*_0_ and *d*_2_, but must travel through *d*_1_ where it is neutral or very weakly selected for. The Perl scripts can be found in the supplement as S1 File.

**Fig 1:**
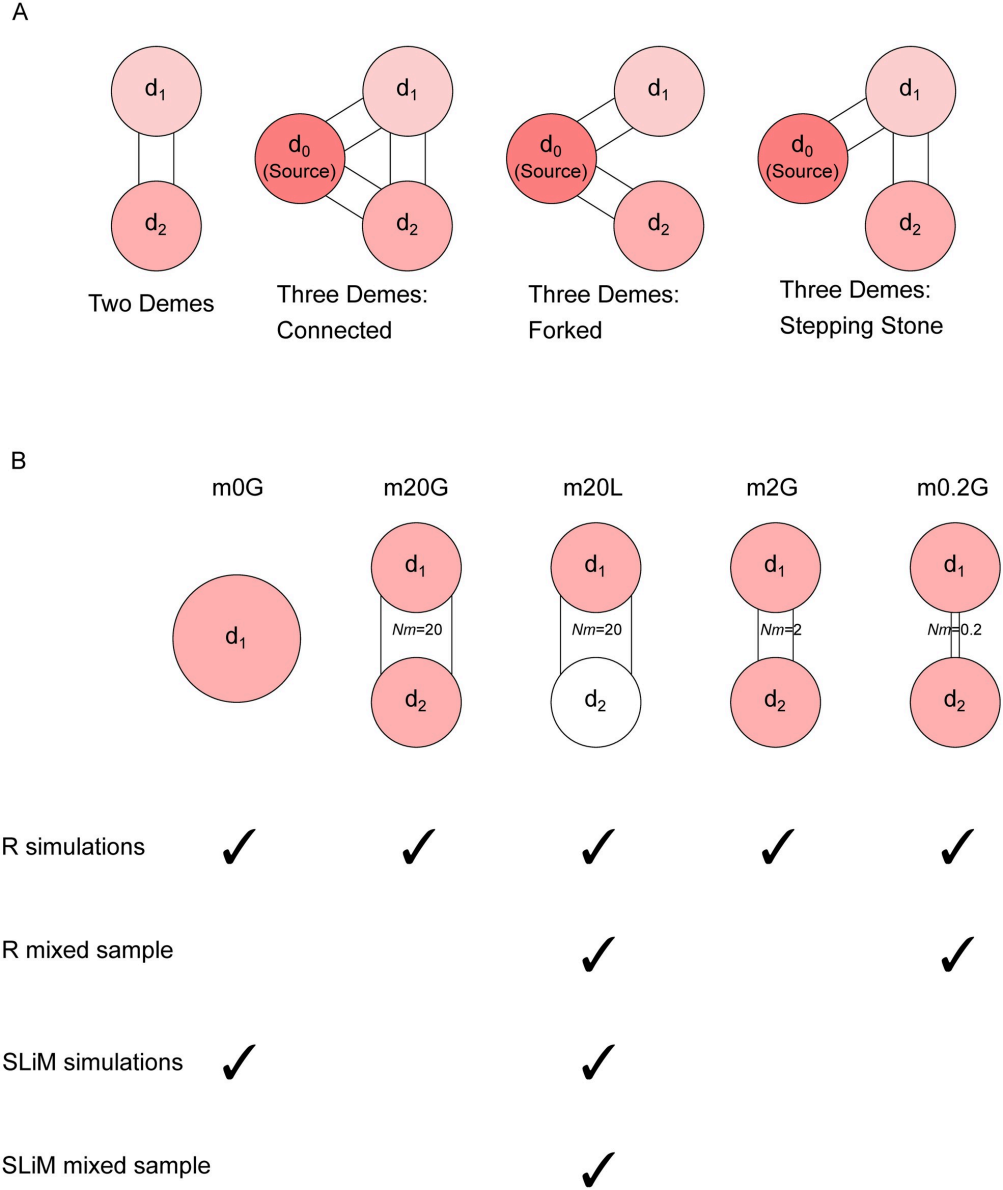
Schematic of evolutionary scenarios in this study. A: Possible demographies for single-site simulation; two-deme and three possibilities for three-deme. Different shades indicate that selection strength *can* be different between demes. Migration rates are identical between deme pairs and range from 0.02 to 200. B: Five scenarios explored in the full-locus simulation. Light red indicating selection in that deme and white indicates neutrality. Only *m*20*L* and *m*0.2*G* were analyzed for mixed-deme samples from R simulations (second row). Only *m*0*G* and *m*20*L* were simulated with SLiM (third row), of which only the latter were analyzed for mixed-deme samples from SLiM simulations (fourth row). Other scenarios listed in Table 2 were used for *F*_*ST*_ analysis only.

Table 1 lists the population sizes and selection coefficients for the one-locus simulations. *N*_*e*1_ and *N*_*e*2_ are the population size (haploid) of each deme, which are 10, 000 individuals unless noted otherwise. The population size of *d*_0_ is always 10, 000. The selective coefficients of the focal allele in demes *d*_0_, *d*_1_ and *d*_2_ are respectively *s*_0_, *s*_1_, and *s*_2_. In all cases we considered five migration rates: *Nm* = 0.02, 0.2, 2, 20 and 200. This wide range of parameters are possible because single-site simulations are much faster than the infinite site model; the results from single-site simulations can therefore guide and motivate the choice of parameters for the full-region simulations. Furthermore, for the three deme model, we consider the following migration graph layouts (Fig 1A): connected, forked, and stepping stone. This leads to a total of 55 parameter combinations for two-deme models and of 120 combinations for three-deme models. For each of these parameter settings we simulated 1, 000 replicates. To reduce the computational load, the deme *d*_0_ trajectories for all *s*_0_ = 0.02 situations were shared. We omitted the cases where *m* = 0.02 (very low migration) and *s* = 0 (no selection) in either deme, because of the exceedingly long waiting-time until fixation in that deme.

**Table 1:** Single-site: List of (haploid) population sizes and selective coefficients. Each row is a scenario that contains 1, 000 replicates for each migration rate. For the scenarios marked with an asterisk, the migration rate *Nm* = 0.02 was not used. *s*_0_, *s*_1_, and *s*_2_ are the selection coefficients for demes *d*_0_, *d*_1_, and *d*_2_ respectively.

Demes	*N*_*e*_ different from 10000	*s*_0_	*s*_1_	*s*_2_
Two	None	NA	0.005	0.005
	None	NA	0.02	0.02
	None	NA	0.05	0.05
	None	NA	0.1	0.1
	None	NA	0.02	0.05
	None	NA	0.02	0*
	None	NA	0.05	0.02
	*N*_*e*1_ = 5000	NA	0.02	0.02
	*N*_*e*1_ = 1000	NA	0.02	0.02
	*N*_*e*2_ = 5000	NA	0.02	0.02
	*N*_*e*2_ = 1000	NA	0.02	0.02
Three	None	0.005	0.005	0.005
	None	0.02	0.02	0.02
	None	0.05	0.05	0.05
	None	0.1	0.1	0.1
	None	0.02	0	0.02*
	None	0.02	0.005	0.02
	None	0.02	0.05	0.02
	None	0.02	0.05	0.05

All population sizes are 10,000 per deme if not specified otherwise.

#### Genomic simulations

Again, we performed forward-in-time simulations with a custom R algorithm. Essentially, we implemented an infinite-sites model, where each new mutation results in a new SNP, uniformly distributed in the region. The population is divided into two demes, each containing *N*_*e*_ = 10, 000 haploid individuals. To simulate a diploid population, we assigned pairs of chromosomes to individuals; the fitness of each individual depends on the diploid genotype.

Recombination is permitted within a pair. The breakpoint for recombination is uniformly chosen along the genomic fragment simulated. The mutation rate of the entire region is *μ* = 0.006 per individual per generation, and the recombination rate is *c* = 0.006 per individual per generation. This corresponds to about 600kb of DNA sequence when the mutation rate is *μ* = 10^−8^ per nucleotide per generation and the recombination rate is *c* = 1*cM*/*Mb*, typically assumed for humans [43], [44]. Migration occurs with probability *m* for each individual and each generation, resulting in an average of *Nm* migrants per generation per direction. A more detailed explanation of the algorithm can be found in S2 File.

The initial equilibrium populations were generated with the program *ms* [45] with the following command line:

$$\begin{array}{c} ms\;20000\;1\;-t\;120\;-I\;2\;10000\;10000\;2*m\;-r\;120\;5000\;-p\;10 \end{array}$$

This produces a neutral population at equilibrium with the same demographic parameters as in our custom R program. From here, an advantageous mutation occurs in one chromosome in one individual in deme *d*_1_ at the start of a simulation cycle. This selected mutation is located at 100kb from the left end of the region. We assume a co-dominant fitness scheme such that wild-type homozygotes have fitness 1, heterozygotes have fitness 1 + *s* and mutant homozygotes have fitness (1 + *s*)^2^ ≈ 1 + 2*s*. We assume further *s* = 0.02. The population was propagated with constant size, and reset to the initial state if the adaptive allele was lost underway.

In models where the population is subdivided, deme *d*_1_ is the origin of the adaptive allele which has a selective coefficient *s* = 0.02; in global adaptation, the allele has the same selective coefficient in deme *d*_2_, while in local adaptation it is neutral in *d*_2_. Seven migration-selection-mutation models were simulated with forward-in-time script (S2 File) each with 100 replicates, and four corresponding neutral scenarios were simulated with the coalescence-based program ms [45] as control. The range of migration rates, from *Nm* = 0.2 to 20 is chosen with two reasons: first, this is the range where most qualitative variation of fixation time was in the single-site simulations (see previous section); second, this is consistent with the range of migration rates estimated from human population data, with *Nm* = 0.2 close to the migration rate between Africa and Eurasia [46], [47], [48] and *Nm* = 20 close to within-Africa rates [49]. We used codes as described in Table 2 to identify the scenarios: the number after “m” indicates number of migrants per generation per direction, and “G” stands for global adaptation while “L” for local adaptation (Fig 1B). “*m*0*G*” is a panmictic population with the size *N*_*e*_ = 20, 000.

**Table 2:** All simulation parameters of two deme model.

Migration Rate	High	Intermediate	Low	Panmictic
*N*_*e*_*m*	20	2	0.2	NA
Global Adaptation	*m*20*G*	*m*2*G*	*m*0.2*G*	*m*0*G*
Replicates	100	100	100	100
Resamples	50	50	50	50
Time points	22	22	22	12
				
Local Adaptation	*m*20*L*	*m*2*L*	*m*0.2*L*	NA
Replicates	100	100	100	
Resamples	50	50	50	
Time points	22	22	200	
				
Neutral	*m*20*NB*	*m*2*NB*	*m*0.2*NB*	*m*0*NB*
Replicates	2500*	2500*	2500*	5000
Resamples	1	1	1	1
Time points	1	1	1	1

Scenarios with selection and neutral control.

*For neutral background of subdivided populations, the two demes are considered two separate samples, effectively resulting in 5,000 replicates. Global (local) adaptation: adaptive alleles is positively selected in both (only one of the) demes. Scenarios *m*2*L* and *m*0.2*L* were used for *F*_*ST*_ analysis only.

Snapshots of the population were recorded at the following time points: (1) when the adaptive allele reached a frequency of 20%, 40%, 60%, 80% and 99.5% in either *d*_1_ or *d*_2_; (2) 1, 000, 2, 000, 3, 000, 4, 000 and 5, 000 generations after the adaptive allele reached 99.5% in either *d*_1_ or *d*_2_; (3) the generation when the adaptive allele fixed in the entire population. A total of 21 snapshots were taken during each simulation run. From each population snapshot, 50 random samples were taken. Each sample contains 50 haploid individuals from *d*_1_ and 50 from *d*_2_. All the analyses and calculations below were done on the *samples* rather than whole populations. For each deme, eleven snapshots were used: partial sweep of 20%, 40%, 60%, 80% and 99.5% *in that deme*, fixation *in the entire population*, and each 1, 000 generations from 1, 000 to 5, 000 generations after reaching 99.5% *in that deme*. In the scenario *m*0.2*L*, the fixation time in *d*_2_ is extremely long, so we took snapshots every 100 generations for 20, 000 generations and then stopped the simulation regardless of fixation.

To obtain the threshold values for the subsequent selection tests, we also simulated neutrally evolving samples with the program *ms*. First, we generated 5, 000 samples of 50 individuals from single-deme populations of size *N*_*e*_ = 20, 000 (m0NB; NB stands for Neutral Background). Second, we generated 2, 500 samples from two-deme populations (*N*_*e*_ = 10, 000 in each deme, *Nm* = 0.2 or 2 or 20; m0.2NB, m2NB and m20NB). Each deme in a sample is used as an independent sample, thus a total of 5, 000 samples of 50 individuals for each migration rate. These neutral datasets are called *neutral background* (NB).

Finally, we also used SLiM 3.2 [50] to simulate additional population samples, to test the effects of increased replicate number and larger sample sizes. Only *m*0*G* and *m*20*L* models are simulated with SLiM as they are representative of our main results (see sections below); 1, 000 replicates are simulated, and 10 samples are drawn from each replicate population (for *m*20*L*, including *d*_1_, *d*_2_ and mixed samples). The sample size is 100 haploid individuals instead of 50. 100, 000 neutral samples are simulated with ms for the panmictic scenario and 50, 000 pairs of neutral samples for *Nm* = 20 scenario. All methods, except evoNet, are applied on these samples with the same method as described below. For details, see S3 File.

### Test statistics for selective sweeps

For scenarios *m*20*G*, *m*20*L*, *m*2*G*, and *m*0.2*G* we calculated 13 different summary statistics at each time snapshot; preliminary analyses showed *m*2*L* and *m*0.2*L* having identical signatures as the global adaptation counterpart in *d*_1_ while behaving entirely neutral in *d*_2_, so they were excluded in the analyses except for population differentiation. These are *θ*_*π*_ (average number of pairwise differences), *θ*_*w*_ (number of polymorphic sites divided by the harmonic number of sample size), *θ*_*h*_ (the sum-of-square of derived allele frequency), Tajima’s *D* [51], Fay and Wu’s *H* [52], number of haplotypes (*n*_*H*_), and the haplotype-based statistics *H*_1_, *H*_12_, *H*_2_/*H*_1_ [42], Nielsen’s parametric composite likelihood ratio (CLR, as implemented in Sweepfinder2 (SF2), [14], [53]), *iHS* [10], [54], *nSL* [55] and *XPCLR* [15]. These methods represent both frequency-spectrum-based and haplotype-based families, and comprise of the most commonly used ones from the past three decades. All the statistics were calculated for sliding windows of 100kb with a step size of 10kb producing 51 windows per sample [10], [56].

For *iHS* and *nSL* every single SNP may take the role as pivot nucleotide, necessitating a modified derivation of the false positive rate. We used a method based on [10], taking the proportion of SNPs in a window that is significant (absolute value of the normalized statistic larger than 2) as the statistic for that window. *iHS* and *nSL* values were normalized with the means and standard deviations (separate for each frequency from 0.06 to 0.94) obtained from the neutral background data. For *XPCLR*, which is a two-population method which requires a control population, we use deme *d*_1_ and deme *d*_2_ as *each other’s* control, to show how its power behaves when both demes are undergoing the same sweep (not applied to model “m20L”). Sweepfinder2 (Nielsen’s CLR) uses grid-based computation instead of a window-based one; we set the distance between two grids as 10kb, equal to the step size for windows.

Of the 13 statistics, *θ*_*h*_, *H*_1_, *H*_12_, *XPCLR*, SF2-CLR and the proportion-of-significance of *iHS* and *nSL* are expected to be higher in selected samples than neutral samples, while the other ones are expected to be lower; hereafter by “beyond (the threshold)” we mean “higher” in former cases and “lower” in latter cases. For each sample and each statistic, we say that the statistic detects a sweep if at least *one* of the first 11 windows (i.e. first 200kb, centered on the adaptive locus) gives a score beyond a threshold (see next paragraph). This is to mimic an experimental data analysis that scans a genome far larger than 600kb. The power is the percentage in 5, 000 samples (100 replicates × 50 samples) detected as positive. We also determined the power when only at least *one* among all 51 windows (600kb) gave a score beyond the threshold.

To control for false positives, we derived the thresholds from the NB data. For each statistic, we separate each NB region into three (non-overlapping) 200kb blocks; the highest or lowest value (depending on the expected direction of change in sweeps) from the 11 windows entirely within a block was used as the representation of the block, giving 15, 000 values; the quantiles for 5%, 1% and 0.1% on the same direction were used as thresholds (Table 1). For thresholds used on entire-region tests, we used the highest or lowest value of all 51 windows instead. In this way, we can ensure that a neutral region has a 5%, 1% or 0.1% chance of being detected as a sweep (false positive rate). Separate thresholds were produced for the three different migration rates, as well as for the panmictic NB.

Finally, we calculated inter-deme differentiation as measured by *F*_*ST*_; from each sample, the mean *F*_*ST*_ value of all sites in the first 200kb was computed. For each replicate and each time stage, the *F*_*ST*_ value used is the mean of all 50 samples. Here all six scenarios *m*20*G*, *m*20*L*, *m*2*G*, *m*2*L*, *m*0.2*G* and *m*0.2*L* were analyzed.

### The detection of selective sweeps with machine learning

We used two machine learning methods, evolBoosting [57] and evoNet [58] to detect selective sweeps by combining multiple summary statistics across sliding windows. A machine learning method does not have a fixed model or algorithm; it must be produced from training datasets (train-sets). To test the effects of different train-sets, we trained the algorithm with backward-simulated *single-deme* hard and soft sweeps in different stages. The simulation was conducted with *discoal* [59], a *ms*-like simulator that can produce samples under different types of selective sweeps. Five stages were examined: (1) an ongoing sweep at 60%, (2) 80%, (3) and 99.5% completion; a sweep (4) at fixation, and (5) at 1, 000 generations after fixation. These train-sets were simulated from a panmictic population of size *N*_*e*_ = 20, 000, with mutation and recombination rates identical to those in the forward-in-time simulations. An additional train-set was produced by pooling a fifth of the data from each stage. Each stage includes 1, 000 hard sweep samples and 1, 000 soft sweep (initial frequency 10%) samples each of 50 haploid individuals. The initial frequency is arbitrarily chosen to establish the difference of hard and soft sweeps. A set of 2, 000 neutrally-evolving samples were simulated with the same demographic conditions as null models in train-sets.

A pair of evolBoosting predictors was trained from each stage to detect and distinguish soft and hard sweeps, and named respectively p60, p80, p99.5, p100, p100+ and pmix. As above, the statistics used in evolBoosting were calculated for a sliding window of width 100kb and step size 10kb. We trained six pairs of predictors with the first 200kb (100kb before and after the selected site), and six pairs with the entire 600kb region. In each predictor pair, the first predictor is produced from 2, 000 neutral samples as a null model (*y* = 0) and 2, 000 sweep samples, hard and soft combined, as alternative model (*y* = 1); the second predictor is produced from 1, 000 soft sweep samples as null model and 1, 000 hard sweep samples as alternative model. A predictor converts the sample into a number, and the higher it is, the more the data is considered “similar” to the alternative model. To control false positives, 5, 000 neutral samples (see section Model and Simulation; independent from the train-set) were tested with the first predictor, and thresholds were chosen to obtain 5%, 1% and 0.1% false positive rates. Thresholds were produced from one-deme and two-deme neutral background samples. For the second predictor (which distinguishes hard from soft sweeps), we used the default threshold (0.5) in all cases, because we want to avoid an *a priori* bias towards either type of selective sweep.

Cross-testing of train-sets was done to assess the sensitivity of sweep detection when train-set and test-set are not synchronized in their stages; hard and soft sweep datasets of all five stages are tested. Testing of our forward simulation datasets are done in the following way: a test-set is derived from either deme *d*_1_ or deme *d*_2_ in a snap-shot stage of a migration-selection model; 100 replicates of sample size 50, i.e. a total of 5, 000 sequences, were produced for a test-set. All test-sets were tested with all six predictor pairs. A sample is classified as “neutral” if it scores below the threshold in the first predictor; as “soft sweep” if above the threshold in the first predictor but below 0.5 in the second; and as “hard sweep” if above the threshold in the first predictor and above 0.5 in the second. The percentage classified as soft sweeps and hard sweeps were recorded for each test-set and each predictor pair.

For evoNet [58], a deep learning algorithm, predictors were trained with the same training sets as above, from samples at 99.5% allele frequency, at fixation, and at 1, 000 generation after fixation. Only 200kb fragments were used. Because multiple-category classification is possible with evoNet, we trained the models with neutral data, hard sweeps and soft sweeps at the same time. The size of the neural network is 8+8 hidden nodes. Using these models, we analyzed all data simulated by R, including *m*0*G*, *m*20*G*, *m*20*L*, *m*2*G*, *m*0.2*G* scenarios. However, because the result is probability-based instead of giving a output statistic, it is impossible to control the threshold with a fixed false positive rate. Therefore, the classification proportions are directly based on the output of the program.

## Results and discussion

### Speed of adaptation by imported beneficial alleles

To determine the speed of adaptation, i.e. the time it takes for a beneficial variant to become common within a deme, we first simulated the trajectory of frequencies of a single adaptive SNP across subdivided populations (single-site simulations, Table 1; see S1 File for the simulation script). We define the “completion” of adaptation as when the allele frequency reaches 99.5%, because the time for the last 0.5% is highly variable with drift dominating this stage. In addition, we also defined the duration of the selection phase as the time interval it takes for the adaptive allele to increase in frequency from 5% to 99.5%. Here deme *d*_1_ refers to the deme in which the adaptive allele originated, and deme *d*_2_ to the one which received the adaptive allele by migration. The detailed results for each scenario can be found in S1 Table.

**Fig 2:**
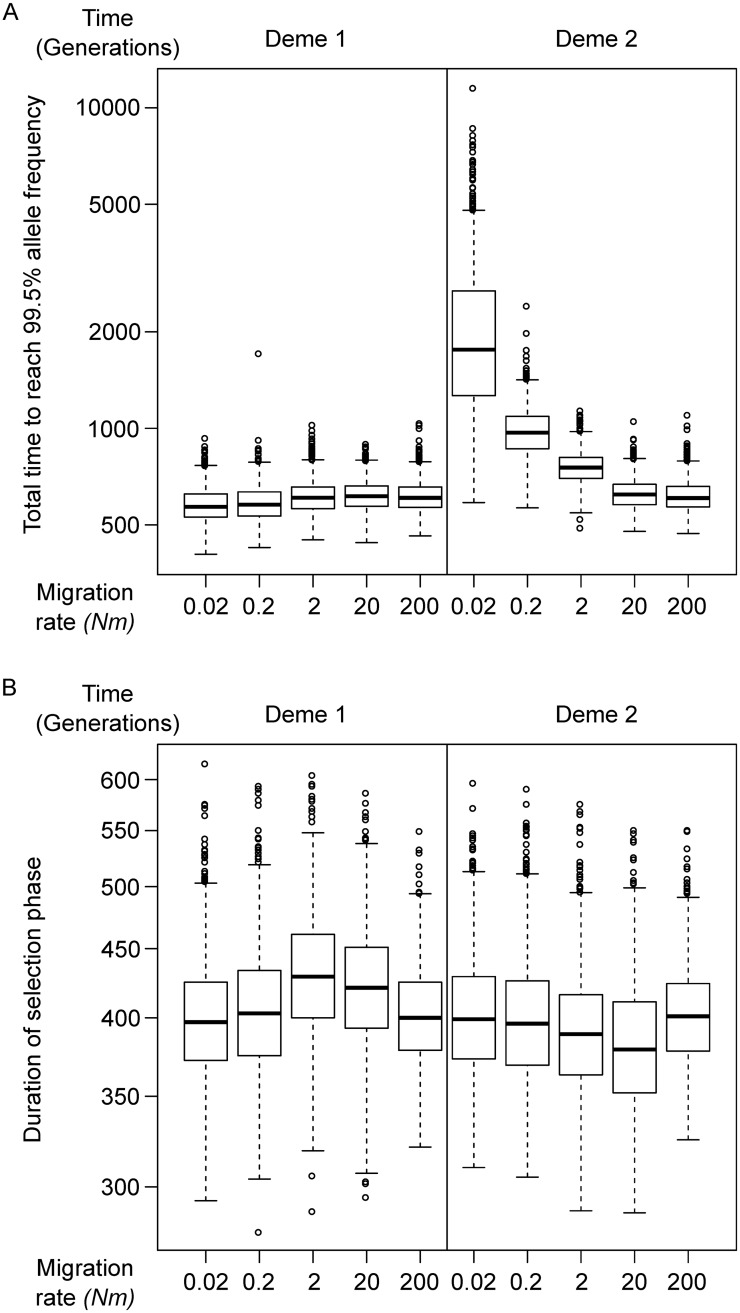
Single-site simulation: Migration rate and time. A: The time taken for an adaptive allele from the beginning (mutation event) to reach a frequency of 99.5%. B: The duration of selection phase, defined as the time between the adaptive allele reaching 5% and 99.5%.

The migration rate, as intuitively expected, strongly affects the time required to reach 99.5% in deme *d*_2_ (Fig 2A; *p* < 0.0001, *r*^2^ = 0.3555, with *Nm* log-transformed). At a migration rate of *Nm* = 20 (chromosomes per generation) or higher, both demes complete their selection phase almost simultaneously. With smaller migration rates, fixation in deme *d*_2_ is delayed compared to deme *d*_1_. However, their relationship is not linear, reciprocal or logarithmic (*Nm* = 2, $\bar{t}=838.5$; *Nm* = 0.2, $\bar{t}=1041.5$; *Nm* = 0.02, $\bar{t}=1861.5$; where $\bar{t}$ refers here to the *median* time to fixation). The duration of the selection phase is largely independent from the migration rate (Fig 2B; *r*^2^ = 0.0039). This implies that migration plays a role only for the first one, or few, adaptive alleles which enter deme *d*_2_, but not for the selection phase itself. The correlation between log *Nm* and the time required to reach 5% in deme *d*_2_ is *r* = 0.3545, almost identical with the correlation between log *Nm* and time to 99.5% as shown above. This is because the time from beginning to 5% is determined by the arrival time of first successful migrant carrying the adaptive allele, while the selection phase is entirely determined by the strength of selection.

In contrast to migration and selection coefficients, population size has only a minor effect on the durations (S1 Fig). The theoretical time length for selection is proportional to *log*(*N*_*e*_)/*s* [60], therefore a 25% reduction for *N*_*e*_ = 1, 000 and a 7.5% reduction for *N*_*e*_ = 5, 000 is expected, compared to a default of *N*_*e*_ = 10, 000. However, an exception occurs for *d*_1_ at an *intermediate* migration rate (*Nm* = 2): the selection phase in *d*_1_ is instead extended when *N*_*e*__1_ is small (S1D Fig; *p* < 0.0001, *r*^2^ = 0.0616), due to unadapted migrants from *d*_2_. If the migration rate is lower, the influx of unadapted migrants is not strong enough to matter (*p* < 0.0001, *r*^2^ = 0.0851); if the migration rate is higher, the allele frequency will be synchronized so that migrants from *d*_2_ are as likely to carry the adaptation as a native *d*_1_ individual (*p* < 0.0001, *r*^2^ = 0.0423). In both of these cases duration of selection is positively correlated with *N*_*e*__1_, consistent with the expectation from a panmictic model. The difference in *total time* for *d*_2_ is negligible because the waiting time for adaptive migrants has high variance and outweighs the effect of varying *N*_*e*_. With high migration rate (S1E and S1F Fig), the differences between different *N*_*e*_ values are even smaller than with an intermediate migration rate. When migration is very high, the entire population can be considered panmictic with *N*_*e*_ = *N*_*e*1_ + *N*_*e*2_; therefore, *N*_*e*_ only varies between 11, 000 and 20, 000, which corresponds to a 6.4% difference in selection time length.

We also simulated the trajectory where *both* demes *d*_1_ and *d*_2_ imported the adaptation from a third deme (deme *d*_0_) which underwent a classic selective sweep. The results for each scenario and parameter combination can be found in S1 Table. In general, the time to fixation is similar to the two-deme situation, as the migration rate dictates when the first adapted migrant become established. Under the “forked” demography, the fixation process is slightly slower due to inability of *d*_1_ and *d*_2_ to exchange migrants, but the difference is tiny even under low migration. This is likely because the situations where one deme received an adaptive migrant far before the other, *and* exported an adapted migrant to the latter, are exceedingly rare. However, the variance of fixation time is increased compared to the “connected” case under the lowest migration rate of *Nm* = 0.02, due to aforementioned rare situations skewing the distribution. For the “stepping stone” scenario, the variance of fixation time in *d*_2_ is very high, while the variation in *d*_1_ is similar to the “forked” case; this can be easily understood as the fixation time in *d*_2_ is the sum of *two* random waiting times.

**Fig 3:**
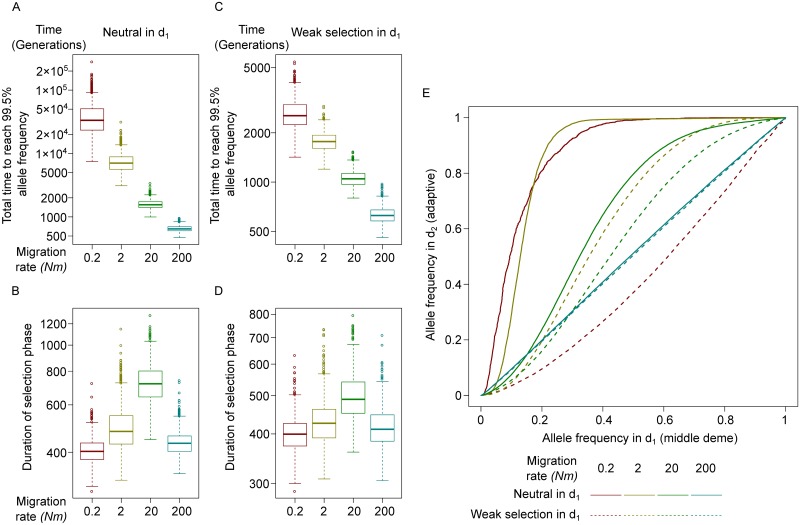
Single-site simulation: Through a middle-deme. A, C: The time taken for an adaptive allele from the initial mutation event to reach a frequency of 99.5% in the destination deme *d*_2_. B, D: The duration of selection phase, defined as the time between the adaptive allele reaching 5% and 99.5%, in the destination deme *d*_2_. The allele has no fitness effect (A, B), or a very weak one (C, D) in the middle deme *d*_1_. E: The average joint trajectory of the adaptive allele frequency in *d*_1_ and *d*_2_, where the allele is neutral (solid lines) or very weakly selected (*s* = 0.005, dash lines) in *d*_1_. In the left-top half, the frequency is lower in *d*_1_. Different colors indicate different migration rates.

A special case for the three-deme model is a stepping-stone migration matrix where the allele is neutral in the middle deme *d*_1_. In this case, the allele must travel through a neutral deme to arrive at deme *d*_2_ where it is again beneficial. The total time, from origination of an adaptive allele in *d*_0_ and fixation in *d*_2_, is logarithmically reduced with increasing migration rates (Fig 3A). However, The length of the selection phase does not behave monotonically (Fig 3B); under the high migration rate of 20 migrants per generation the selection phase is longer than under a higher *or* lower migration rate. When migration rate is *Nm* = 200, there is practically no distinction between *d*_1_ and *d*_2_, thus the process is equivalent to selection in a population twice as large under half the selective coefficient. However, when *Nm* increased from 0.2 to 20, the allele has consistently a higher frequency in *d*_2_ than *d*_1_, meaning that migrants from *d*_1_ are less likely to be adapted than an average individual in *d*_2_. See Fig 3E for the joint frequency trajectory in *d*_1_ and *d*_2_. If the allele is not neutral in the “middle deme” but only slightly positively selected (*s* = 0.005), the total time until fixation in *d*_2_ can be extremely shortened (Fig 3C), but the non-monotonic behavior still persists (Fig 3D). In other words, a neutral middle deme can serve as a barrier even though selection occurs in both demes connected with it.

### Selective sweeps in the panmictic model

We describe now in detail the results from our genomic-region simulation experiments (see S2 File for the simulation script), starting with the panmictic scenario in this section. Power is the percentage of the 5,000 samples (100 replicates each giving 50 samples) detected as being under selection, based on a threshold which corresponds to a 1% false positive rate on neutral background samples. While false discovery rate (FDR) is a common measure of a detection method’s accuracy, it requires a dataset in which positive and negative instances occur in “natural” proportions. Our data do not meet this criterion, because the relative sizes of selection and neutral datasets are arbitrarily decided. For *each* deme, twelve time points were tested: neutral (before selection starts), adaptive allele frequency within deme reaching 20%, 40%, 60%, 80% and 99.5% (abbreviated below as $f_{20}^{s}$, $f_{40}^{s}$, $f_{60}^{s}$, $f_{80}^{s}$, $f_{99.5}^{s}$,); 100% in the entire population (global fixation, $f_{100}^{s}$); 1, 000, 2, 000, 3, 000, 4, 000 and 5, 000 generations after $f_{99.5}^{s}$ ($t_{1K}^{r}$, $t_{2K}^{r}$, $t_{3K}^{r}$, $t_{4K}^{r}$, $t_{5K}^{r}$). A total of 5, 000 parallel samples (100 independent populations, and 50 samples from each) were produced from *each* scenario, *each* deme and *each* time point.

**Fig 4:**
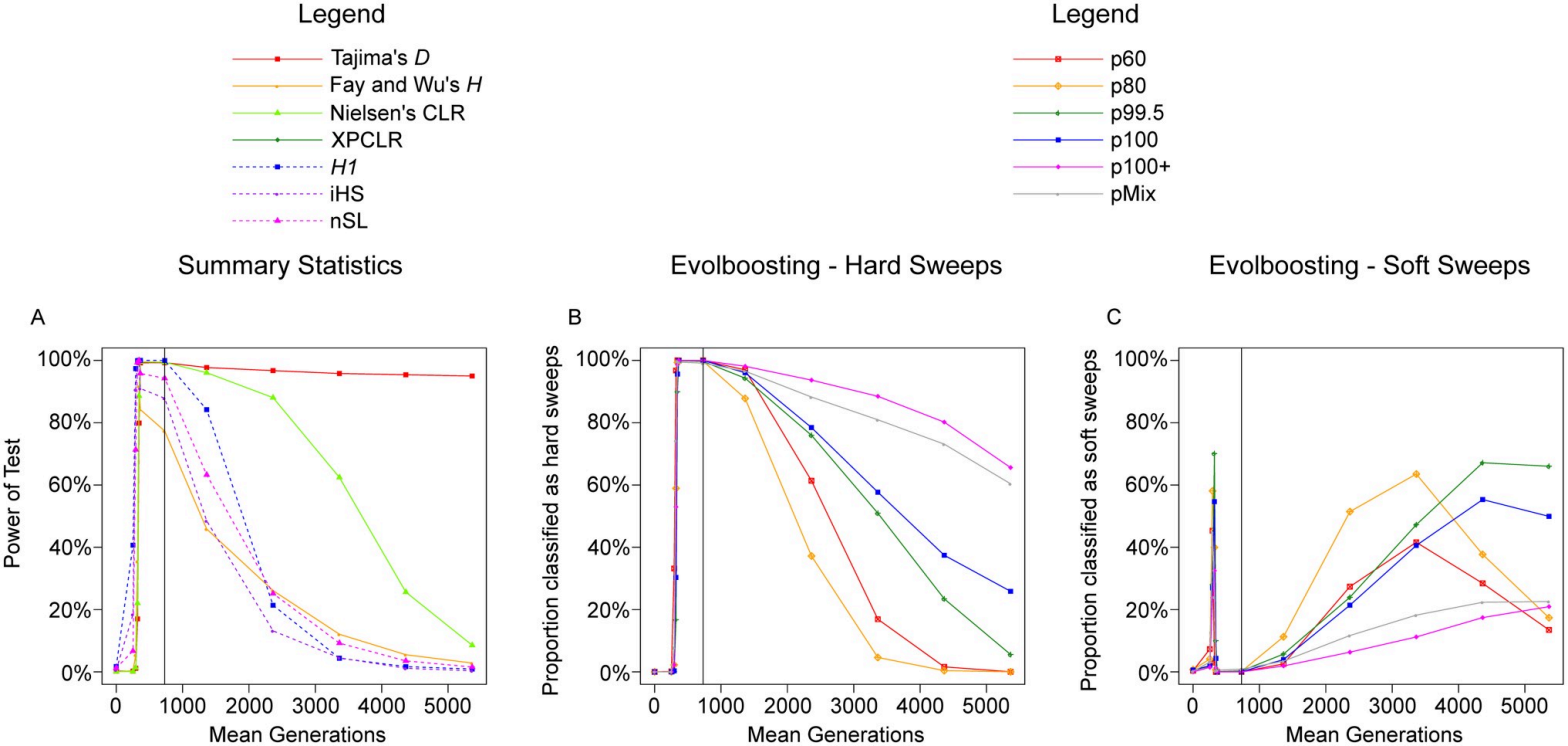
Full-locus simulation: The detection rate of various methods in a panmictic scenario. The proportion of samples detected as selective sweeps by various methods, under the scenario *m*0*G*. The vertical line indicates time of 100% fixation. A. Power of seven summary statistics; dashed lines indicate haplotype-based methods. B. Proportion detected by six EvolBoosting predictors *correctly* as hard sweeps. C. Proportion detected by six EvolBoosting predictors *incorrectly* as soft sweeps. See S3 Fig for a zoomed-in version for early stages.

First we observed the performance of summary statistics in a panmictic population (Fig 4A). The methods can be separated into two classes. Those that are based on the site frequency spectrum, including Tajima’s *D*, Fay and Wu’s *H* and Nielsen’s *CLR*, reach high power at $f_{80}^{s}$ and stayed high until $t_{3K}^{r}$ (Except *H* which loses its power faster). In contrast, methods based on haplotype length and structure, such as *H*_1_, *iHS* and *nSL*, already have very high power at $f_{20}^{s}$ to $f_{40}^{s}$, but lose it by $t_{2K}^{r}$. (Number of haplotypes, *H*_12_ and *H*_2_/*H*_1_ are not shown because they are almost identical to, or of lower power than, *H*_1_.) In other words, haplotype-based methods excel with ongoing and early-stage sweeps while frequency-spectrum-based ones are more powerful for completed sweeps. This is consistent with the fact that the methods such as *iHS* are designed for ongoing sweeps rather than for completed ones.

The machine learning algorithm EvolBoosting combines multiple summary statistics to distinguish between pre-determined scenarios [61], [57]. We trained six pairs of predictors, each containing one predictor capable of detecting sweeps from neutrality and one to distinguish hard sweeps from soft sweeps. The train-sets are simulated with panmictic populations with *N*_*e*_ = 20, 000. Five pairs of predictors are based on sweeps in different stages, while the sixth derives from a mixture of five stages. These predictors are called p60 ($f_{60}^{s}$), p80 ($f_{80}^{s}$), p99.5 ($f_{99.5}^{s}$), p100 ($f_{100}^{s}$), p100+ ($t_{1K}^{r}$) and pMix (mixture). A predictor converts the statistics from a region into a single number; the larger it is, the more “sweep-like” the region is determined to be. We derived cut-off values for sweep detection from controlling false positive rates (*α* = 0.05, 0.01 or 0.001) on samples of neutrally evolving regions; these samples are same as the neutral background mentioned in the previous section, and independent from the train-sets.

**Fig 5:**
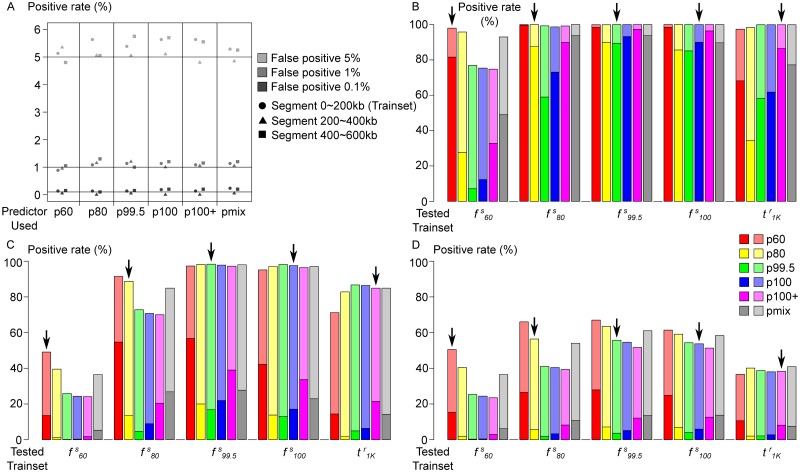
Full-locus simulation: Cross-testing EvolBoosting predictors in simulated panmictic populations. A: False positive of neutral data, where only the segment 0–200kb (circles) is used as training sets. Similarity among the three segments indicate absence of over-fitting. B-D: The proportion of samples detected as soft (lighter color) or hard (darker color) selective sweeps, using each other’s training sets for testing. B: Cross-testing using hard sweep samples of different stages, using the region within 100kb from selection site. C: Cross-testing using soft sweep samples of different stages, using the region within 100kb from selection site. D: Cross-testing using hard sweep samples of different stages, but using the region 100–300kb away from selection site. The down-arrow indicates where the tested dataset matches the stage of predictor.

Fig 5 shows the result of cross-testing, i.e., using each other’s train-sets to test the predictors. The risk of over-fitting is limited by comparing the (false) positive rate from the three 200kb parts of neutral regions (Fig 5A), in which only the first part is involved in training. Fig 5B shows how hard sweep regions were classified, which is determined by an interaction between time-stage of the test set and the time-stage on which the predictors were trained. With the exception of $f_{60}^{s}$, all train-sets are identified by all predictors as sweeps, in which a proportion was misclassified as soft sweeps. In particular, more than half of the samples with $f_{60}^{s}$ were misclassified by all predictors except p60, and $t_{1K}^{r}$ samples were misclassified by early-stage predictors. Surprisingly, p60 is the best-performing predictor for all datasets, except for $t_{1K}^{r}$. This is likely because a sweep’s signature gradually increase with adaptive allele frequency, thus p60, trained on weaker signals, is most sensitive. Generally, we observe a tendency to mis-classify hard sweeps as soft when there is a stage mismatch between train- and test-sets of a predictor. We call this effect *temporal softening*. The same phenomenon is observed from the results of evoNet, a deep learning algorithm for selective sweep classification [58] (S2 Fig); misclassification as soft sweeps is common at both ends of the timeline but rare at time stages close to fixation.

We observe similar patterns when the entire 600kb region was used to train the predictors instead of the 200kb fragment around the selection site (S2 Table). While cross-testing only contains the result between $f_{60}^{s}$ and $t_{1K}^{r}$, Fig 4B shows the power of the predictors throughout all time stages. Early on during the selection phase, p60 gains power the fastest as expected (Fig 4C; for the details see S2 Table and S3 Fig). For stages later than $t_{1K}^{r}$, power of different predictors decays at different speed. As expected, power of later-stage predictors remains long after fixation. For instance, p100+ retains over 60% power for hard sweeps (over 80% for hard + soft) at $t_{5K}^{r}$. Concomitant with a decay of power, especially for early-stage predictors, such as p60 and p80, we observe a strong tendency for softening (Figs 5B and 4C). In conclusion, temporal softening occurs on both ends of the sweep process. By using only one type of predictor it is very difficult, if not impossible, to make a reliable distinction between hard and soft sweeps. For sweeps of unknown time-stage, it would be more prudent to use both early- and late-stage predictors jointly.

An opposite effect can be observed when some of the predictors are used on soft sweep samples (Fig 5C). Among all six predictors and in our tested datasets except for $t_{1K}^{r}$, p60 is most likely to classify a soft sweep sample as hard. The predictors p100+ and pmix also mis-classify soft sweeps as hard. Note that, unlike in the situation with hard sweeps, the best and worst predictors are consistent, instead of a complex interaction between predictor and test-set. This phenomenon can be similarly called “temporal hardening”; collectively, “temporal misclassification” denotes the classification of hard sweeps as soft, and soft sweeps as hard, when the training model and tested data mismatch in time stage. Additionally, we trained predictors only with soft sweeps, and cross-tested them on the same soft sweep training sets. The percentage detected with soft-only predictors are almost identical with the hard + soft predictors, with the exception that soft sweeps at $f_{60}^{s}$ are better detected by soft-only p80, p99.5 and p100 predictors by 5 to 10% (S2 Table).

Finally, a decay of sweep detection power based on *distance* from the selection site can be easily observed. The 600kb region was split into three 200kb ones, and the selection site is at the center of the first of them. When we use the predictors on the second region (0.1 to 0.3 cM from selection) instead, the “soft shoulder” effect [21] can be seen as the region *next to* a hard sweep can be recognized as a soft sweep (Fig 5D). Similar to “temporal softening”, the p60 predictor was the least affected. In the third (more distant; 0.3 to 0.5 cM from selection) 200kb region predictors have a lower detection rate (highest is 6–8% for $f_{99.5}^{s}$) and almost exclusively detected as soft sweeps.

Increasing the sample size from 50 to 100 haploid individuals caused the power of most methods to be marginally improved; see S3 File for details.

Classification of sweeps as “hard” and “soft” often relies on ideal assumptions such as known time stage and genomic location of the selection site, as well as demographic assumptions such as a panmictic population of constant size. In regard to the location-based effect known as “soft shoulder”, potential solutions include explicitly modeling regions linked to hard sweeps as well as classify sweeps based on signal peaks only [21], [20]. On the contrary, our “temporal softening” is caused by an early-stage hard sweep mimicking the signal of later-stage soft sweeps: multiple haplotypes at locus, weaker reduction of genetic diversity, and a one-peak patterns for statistics like Fay and Wu’s *H* or linkage-based ones [20] (two-peak patterns occur for fixed hard sweeps). The peaks refer to the shape of the statistics along the chromosomes, surrounding the site of the adaptive allele.

When a machine-learning algorithm is trained with sweeps of only one time stage, or a statistic (especially a likelihood-ratio test) is created based on only ongoing or fixed sweeps, it can be unable to recognize patterns for other stages; this is independent of *which* type of algorithm it is based on. Most studies before have been focusing on only ongoing [62] or fixed [21] sweeps. So far, little attention has been paid to the question of how robust the tools are with respect to stage mismatch and how much false positive and negative rates may be inflated by this problem. We thus argue that searches for sweeps in genomic data, especially those that also try to distinguish hard and soft sweeps, need to explicitly account for the different stages (ongoing, recent or ancient) in the models and (if applicable) machine-learning training sets. One caveat, though, is that our soft sweep training sets were simulated using 10% as the initial frequency, to increase the probability that there are multiple haplotypes carrying the adaptive allele in the population. If the number is reduced, for example, to 0.75% (the median allele frequency in a population with *N*_*e*_ = 10000, instead of the mean), a larger number of soft sweeps would be indistinguishable from hard sweeps, reducing the rate for correct classification.

In our simulation studies here we did not explicitly include incomplete sweeps that stopped (became neutral) before reaching fixation. This can be caused by either environmental change or frequency-dependent selection. If caught when selection ceases, they should behave in the same way as ongoing hard sweeps—a case we did consider. When the allele loses the advantage before a strong sweep signal has built up, the deterioration of the signal from this time point onwards will more quickly render it undetectable, compared to completed sweeps.

### Global selective sweeps in a sub-structured population

For a population with subdivision into two demes we study how sweep detectors are affected by migration and which differences, if any, exist between the deme where the adaption is native to *d*_1_ and the one it is imported into *d*_2_. Our scenarios include global selection with low, intermediate and high migration, and local selection (see the following section) with high migration (abbreviated “*m*0.2*G*”, “*m*2*G*”, “*m*20*G*” and “*m*20*L*”, respectively, where the number indicates the migration rate per generation per direction). Deme *d*_1_ is the origin of the adaptive allele which has a selection coefficient *s* = 0.02; in global adaptation, the allele has the same *s* in deme *d*_2_, while in local adaptation it is neutral in *d*_2_. Samples were taken during the selection phase as well as after fixation.

**Fig 6:**
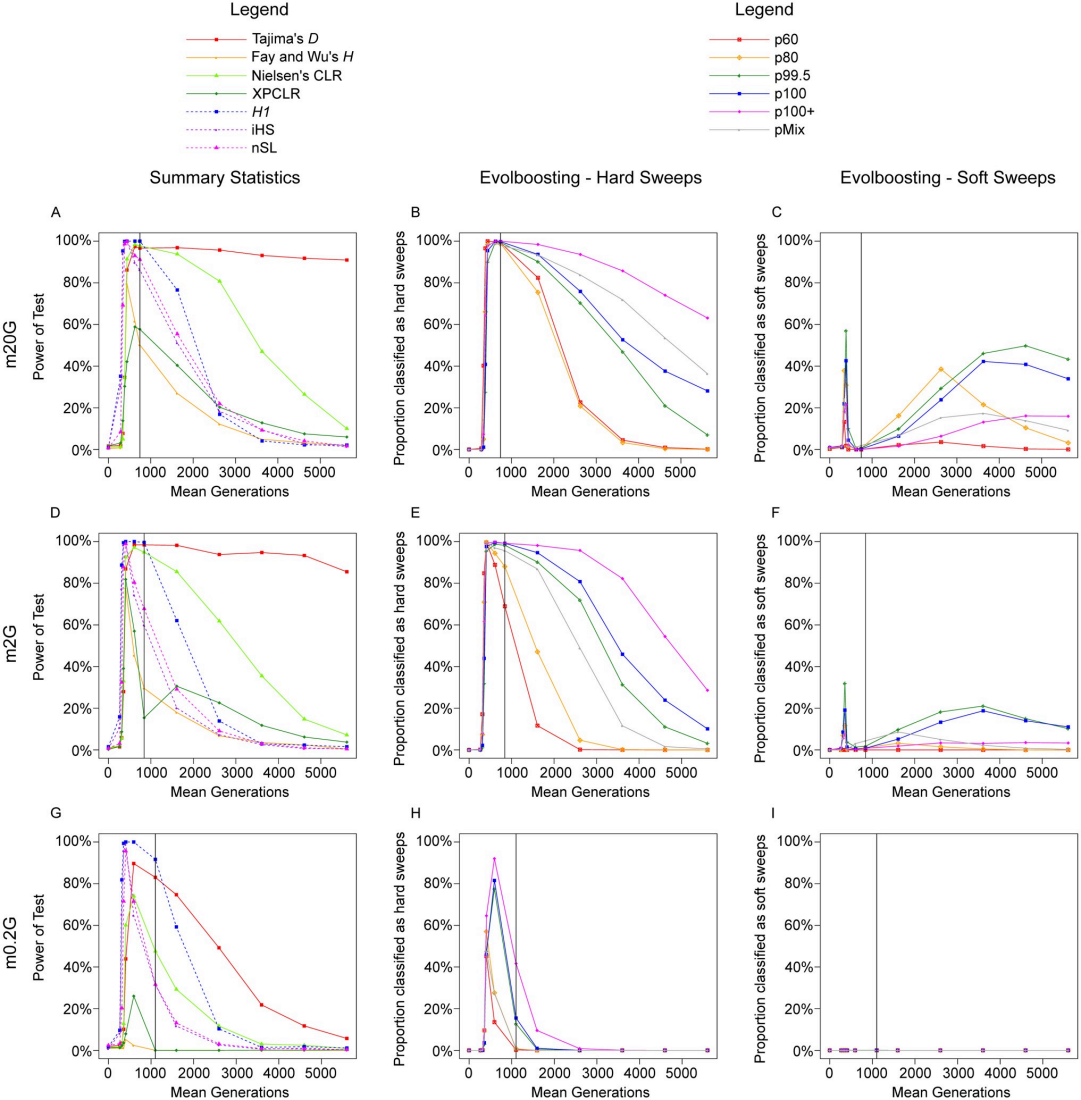
Full-locus simulation: The detection rate of various methods in global adaptation scenarios in the native deme. The proportion of samples detected as selective sweeps by various methods, under the scenarios: A-C. *m*20*G*, D-F. *m*2*G*, G-I. *m*0.2*G*, in *d*_1_ where the adaptive allele arises. The vertical line indicates time of 100% fixation. A,D,G. Power of seven summary statistics; dashed lines indicate haplotype-based methods. B,E,H. Proportion detected by six EvolBoosting predictors *correctly* as hard sweeps. C,F,I. Proportion detected by six EvolBoosting predictors *incorrectly* as soft sweeps. See S3 Fig for a zoomed-in version for early stages of *m*0.2*G*.

When migration is high (*m*20*G*) or intermediate (*m*2*G*), the overall situation is similar to the panmictic case (Fig 4A). Fig 6A, 6D and 6G shows the results from summary statistics in *d*_1_; the results from *d*_2_ is similar (S4A, S4D and S4G Fig). Both frequency-spectrum-based and haplotype-based methods, except *XPCLR*, reach a very high power (80% or higher) peaking at around $f_{80}^{s}$ to $f_{99.5}^{s}$. Compared to the panmictic scenario, Fay and Wu’s *H* has reduced power particularly for *m*2*G*. In addition, the power of *iHS* and *nSL* decays before the adaptive allele has globally fixed.

The cross-population comparison method *XPCLR* assumes (1) selection occurs only in the focal deme and not in the control deme, and (2) there is no migration between the demes and they have recently diverged. For global adaptation in populations with migration, both assumptions are violated in our study. Under such a condition, XPCLR is *not* a reliable method for sweep detection. This incorrect application leads to the lowest power among the tested statistics. *XPCLR* performs similarly in both demes under *m*20*G*, but much better in *d*_1_ than *d*_2_ under *m*2*G*. This is because when the adaptive allele reaches high frequency in *d*_1_ but not in *d*_2_, the latter seems more “neutral”. Interestingly, the power of *XPCLR* slightly increases in both demes *after* global fixation under *m*2*G*.

While EvolBoosting predictors were trained with one-deme *N*_*e*_ = 20, 000 samples, the thresholds for sweep detection can be adjusted based on two-deme neutral background samples. For high migration (*m*20*G*) (Fig 6B and 6C; S4B and S4C Fig), the general pattern is similar to the panmictic model (Fig 4B and 4C), except for slightly reduced power at $f_{40}^{s}$ in all predictors and earlier power decay after fixation in early-stage predictors. For intermediate migration (*m*2*G*) (Fig 6E and 6F; S4E and S4F Fig), fast loss of power by early-stage predictors is more clear. Comparing to a panmictic model, the following can be observed: (1) at $f_{60}^{s}$, late-stage predictors are more likely to detect a hard sweep; however the total detection power (hard and soft) is lower. (2) p60 performs much worse in *m*2*G* than under panmixia both in detection window and power. On the other hand, after fixation, the datasets detected as selection are less likely to be misclassified as soft in *m*2*G* than under panmixia (Fig 5B). In both *m*20*G* and *m*2*G*, the difference between *d*_1_ and *d*_2_ is slight. However for *m*2*G*, the power of all predictors are reduced in *d*_2_ at early ($f_{60}^{s}$) and late ($t_{1K}^{r}$ to $t_{3K}^{r}$) stages with slightly increased misclassification as soft.

When migration rate is lower (*m*0.2*G*), all sweep-detection methods suffer a reduced power and/or a smaller detection window (Fig 6G, 6H and 6I; S4G, S4H and S4I Fig). For instance, power of Tajima’s *D* quickly drops after fixation. While *iHS* and *nSL* still have a peak power near 100%, it lasts very briefly; in comparison, only *H*_1_ has power over 80% at global fixation, and performs better than all other methods at this time point. Fay and Wu’s *H* and *XPCLR* are virtually unable to detect any sweeps in any stage. In addition, the dynamics of detection power through time are different between the two demes. Frequency-spectrum-based methods, particularly Tajima’s *D*, has clearly lower power in deme *d*_2_ compared to *d*_1_. On the contrary, haplotype-based methods are better with *d*_2_. In general, low migration between demes has less adverse effects on the power of haplotype-based methods than of frequency-spectrum-based ones.

Under low migration, the detection rate by EvolBoosting predictors is also reduced. All predictors have little power before $f_{60}^{s}$ and after fixation, particularly p60 and p80. In late selection phase, p99.5, p100 and p100+ have reasonable power. Interestingly, we do not observe any temporal softening; all detected sweeps are classified as hard. Because of the higher thresholds needed to achieve the same false positive rate, only the most “obvious” sweeps are detected; these are also sweeps that have signals that match best “hard sweeps” in the training set. The samples that would be caught in temporal softening became false negatives instead. The patterns for deme *d*_1_ and *d*_2_ over time are slightly different; at global fixation, detection power is almost lost in *d*_1_ but not *d*_2_, because *d*_1_ is already recovering from the sweep by that time. In general, the lower the migration rate is, the narrower becomes the time window during which the sweep is detectable, and the stronger becomes the difference between *d*_1_ and *d*_2_. Sweeps in deme *d*_1_ need to be detected in an earlier stage than in deme *d*_2_.

We want to point out that these results are also contingent on the false positives being controlled with neutral regions of the *same demographic scenario*. If the neutral null model is panmictic (i.e., incorrect demographic assumptions), all thresholds will become more lenient, leading to rampant false positives under low-migration (see S2 Table). This is expected as a panmictic population with *N*_*e*_ = 20, 000 is equivalent to a two-deme population each of size *N*_*e*_ = 10, 000 where the migration rate is *m* = 0.5, which is numerically closer to our high migration scenario. Among the methods, *iHS* and *nSL*, and to some extent Tajima’s *D*, are less affected by such false positives; particularly *iHS* is almost unaffected.

Population subdivision with limited migration is an important modification of the panmictic-equilibrium assumption in population genetics. We demonstrated that frequency-spectrum-based, haplotype-based and machine learning methods are capable of detecting hard sweeps in subdivided populations, but their power and detection window (the time window in which a sweep can be detected) are reduced in low-migration scenarios. However, with population subdivision, detection power of haplotype-based methods is generally higher across a wider range of migration rates, including low migration, than of frequency-spectrum-based ones. Still, haplotype methods have little power to detect sweeps after fixation. Together with the limited power of frequency spectrum methods under subdivision, this severely limits the ability to reliably detect—in practice—anciently completed sweeps in subdivided populations.

There are two main reasons that lead to reduced sweep detection power under low migration. First, subdivided populations with low migration produces a neutral frequency spectrum that already looks like sweeps. These include an excess of rare alleles brought by rare migrants leading to a negative Tajima’s *D* and lower haplotype diversity than panmictic populations with the same *θ*. In the case of Fay and Wu’s *H*, the signal is diluted by high-frequency derived alleles in neutral background, caused by a few migrant chromosomes carrying the ancestral allele into a deme where the derived one is otherwise fixed. High-frequency derived alleles should be very rare in panmictic neutral datasets, thus a small increase by migration can strongly affect the result. We observed a very high false positive rate if thresholds were controlled with a panmictic null model. Similar results are observed with evoNet (S2 Fig), where close to half of the neutral samples are incorrectly labeled as soft sweeps. By controlling false positives using neutral regions of the same demography, this can be avoided; in biological data this translates to using the genomic data of the samples instead of theoretical parameters. Second, the between-loci variation in such populations is much larger than in high migration or panmictic populations, leading to more stringent thresholds if the same false positive rates are desired, thus reduced power. This is mainly because different loci have different numbers of chromosomes carrying migrant haplotypes. A migrant allele enters the deme with a very low frequency, and can be rapidly lost in a few generations or increase to a intermediate frequency by drift. Sampling error (50 from a deme of 10, 000) exacerbates this problem. Compared to a locus without migrant haplotypes, a locus with one migrant haplotype would have a deeper coalescent tree and more singleton alleles; a locus with *i* migrant haplotypes has an excess of alleles with the frequency *i*/*N*_*e*_.

Finally, a slight but visible difference exists between deme *d*_1_, where the adaptive mutation arises, and deme *d*_2_, where the mutation is imported to. The difference in a high migration scenario is negligible, because the demes are homogenized very quickly and the haplotypes are readily shared. With low migration, a reduction of power in deme *d*_2_ is observed for frequency-spectrum-based methods, especially at $f_{60}^{s}$ to $f_{80}^{s}$. A possible reason is that when the arriving migrant alleles (and haplotypes by extension) increase their frequency to 60–80% and the rest are alleles that are originally in *d*_2_, an *increase*, instead of decrease, of intermediate-frequency alleles destroys the signals that are usually picked up by statistics such as Tajima’s *D* or Fay and Wu’s *H*.

### Local selective sweeps in a sub-structured population with high migration

**Fig 7:**
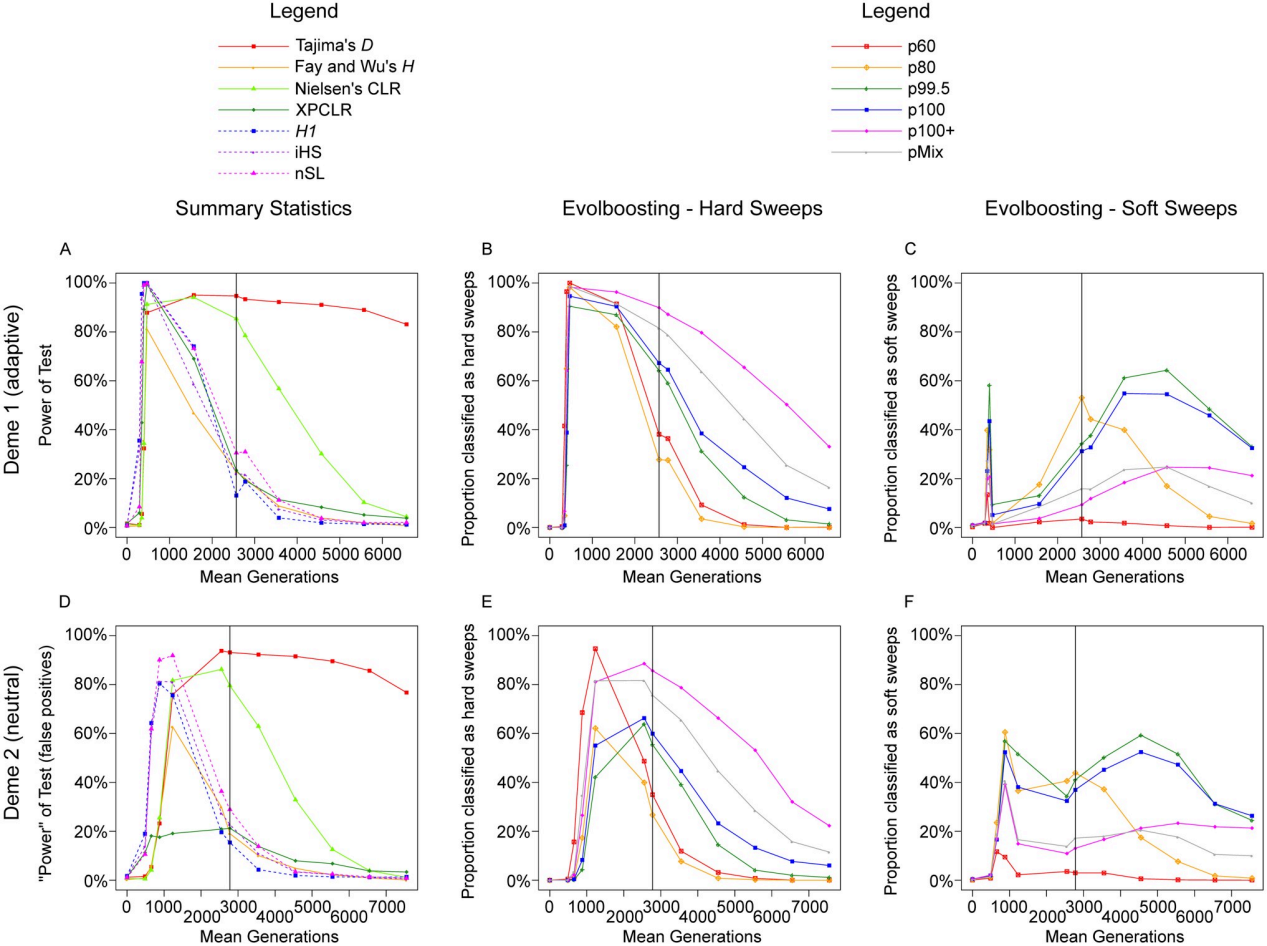
Full-locus simulation: The detection rate of various methods in a local adaptation scenario. The proportion of samples detected as selective sweeps by various methods, under the scenario *m*20*L*. The vertical line indicates time of 100% fixation. A. Power of seven summary statistics in *d*_1_; dashed lines indicate haplotype-based methods. B. Proportion detected by six EvolBoosting predictors *correctly* as hard sweeps in *d*_1_. C. Proportion detected by six EvolBoosting predictors *incorrectly* as soft sweeps in *d*_1_. D. *False* positive rate of seven summary statistics in *d*_2_. E. Proportion detected by six EvolBoosting predictors *incorrectly* as hard sweeps in *d*_2_. F. Proportion detected by six EvolBoosting predictors *incorrectly* as soft sweeps in *d*_2_. See S3 Fig for a zoomed-in version for early stages.

In the *m*20*L* scenario (Fig 7), the selective sweep is limited to deme *d*_1_, while the derived allele is neutral in deme *d*_2_. In such cases, migration from deme *d*_1_ would lead to an eventual fixation of the derived allele in *d*_2_. With a sufficiently high migration rate, the time scale of fixation is similar to that of the global adaptation regime, resulting in a sweep-like genetic signature in *d*_2_, i.e., a false positive. With lower migration rates, fixation in *d*_2_ may take too long to manifest as a sweep signal. We analyzed the behavior of summary statistics and of EvolBoosting to examine the feasibility of identifying local adaptation under the high migration model, and the extent of such false positive detections.

Methods based on the frequency spectrum performed generally similar between global and local adaptation, with the exception that Fay and Wu’s *H* is much less powerful around the time of global fixation. This is likely because recombination during the long pre-fixation phase breaks up close linkages, resulting in less high-frequency derived alleles near the adaptive site. Haplotype-based methods show a clear difference between global and local adaptation. The haplotype-based methods, *H*_1_, *iHS* and *nSL*, lost the most power at fixation as the longer time needed broke up the haplotypes. In the non-adapting deme *d*_2_, *H*_1_, *iHS* and *nSL* has peak (false) detection rate of 80% at $f_{60}^{s}$ (S3 Fig). In other words, haplotype-based methods could distinguish global and local adaptation if the tests are done in *d*_2_, particularly if caught slightly before fixation; however the neutral deme could still be tested positive for selection earlier on at $f_{60}^{s}$ or $f_{80}^{s}$. *XPCLR*, being a cross-deme comparison, has very high power in *d*_1_ while lower than 20% in *d*_2_.

Between $f_{60}^{s}$ and $t_{2K}^{r}$, most EvolBoosting predictors can correctly detect a large proportion of *d*_1_ samples as sweeps, although at a lower proportion than under global adaptation. There are considerable differences among the predictors in their (false) positive rate and classification results. Power of p60 degrades quickly after fixation and sweeps are almost always classified as hard. In contrast, p80, p99.5 and p100 have a relatively high false positive rate and classify a large proportion of samples as soft sweeps. This is the case in all stages in deme *d*_2_. But they are correctly identified as hard at $f_{80}^{s}$ and $f_{99.5}^{s}$ in deme *d*_1_.

In deme *d*_1_ additional differences between the global and local selection regimes can be observed. For instance, p60 has a reduced detection window in the *m*20*L* scenario. Furthermore, p80, p99.5 and p100 classify 30–50% of the samples incorrectly as soft sweeps at fixation and at $t_{1K}^{r}$, while the classification is mostly correct (hard sweeps) under the m20G regime. Similarly, p100+ and pmix mis-classify sweeps as soft at $t_{2K}^{r}$ to $t_{5K}^{r}$ much more often under the *m*20*L* regime than under the *m*20*G* regime. We call this effect “*spatial softening*”. While in *m*20*G* sweeps are usually correctly identified as hard in both demes, in *m*20*L* it is likely that it is correctly detected as hard in *d*_1_ but soft in *d*_2_ where it should be neutral. Spatial softening can be also observed with deep learning algorithm evoNet (S2 Fig), where a larger proportion of samples are misclassified into soft sweeps in both demes, for scenario *m*20*L* compared to *m*20*G*; this occurs across almost all time stages.

Additionally we simulated the *m*20*L* scenario with SLiM [50], with 1, 000 replicates and a sample size of 100 haploid individuals per deme. Compared to the sample size of 50, we observed an improvement of power across time, i.e. power was higher in more windows than in the scenario with a smaller sample size. However for EvolBoosting, misclassification as soft sweeps (spatial softening) is also increased. See S3 File for details.

Here we attempt to explain the different results between *m*20*G* and *m*20*L* scenarios. First, we noticed that the *first* deme, where selection always occurs, has a delay in the case of local adaptation. This is because the inflow of unadapted migrants reduces the frequency of the adaptive allele every generation. Second, haplotype-based signals degrade in an earlier stage for local adaptation, compared to global adaptation. This is because a long period of the fixation process is spent when selection is mostly completed in deme *d*_1_, while the frequency of the new allele is increasing only via migration in deme *d*_2_. During this extended period haplotypes are broken up by recombination.

*XPCLR*, the only cross-deme comparison method is able to find the origin of the adaptive allele in a local selection scenario, but fails to efficiently identify such cases in global selection. This is likely because its “control” population is also under selection too, so a strong contrast cannot be produced. We showed that *XPCLR*’s assumption about recently diverged but isolated demes can be relaxed. However, if the *XPCLR* comparison is done with a neutral deme importing a selected allele and *another* neutral deme further distant from selection, it is still possible to falsely detect selection in the former neutral deme [30].

Ideally, a good test should not return a signal for deme *d*_2_ in a local adaptation scenario, because no selection has occurred there. However, the rapid importing of selected migrant alleles shortens the coalescent trees and produce sweep-like genetic footprints. If migration is even higher, such a case eventually converges to a Levene model, where all demes are completely mixed at the reproduction stage. Theory suggests that selection in a Levene model can be approximated by the average level of selection of each deme [63], [64]. In our study, all methods, except *XPCLR*, return high “power” for positive selection in deme *d*_2_ of m20L. In addition, EvolBoosting analyses classified a substantial proportion of such imported “sweeps” as soft sweeps. Natural populations, including human populations, are often characterized by a complex spatial distribution and heterogeneous selective pressure. Therefore, it is possible that the large amount of “soft sweeps” discovered from the human genome [36], [35] are “sweeps by proxy”, i.e. hard sweeps occurring in other populations imported by migration.

To solve this conundrum, we need either a method that explicitly takes into account imported alleles that were fixed by selection in their source deme, or try to sample as many demes as possible and find the source of such alleles by pairwise comparison methods, such as *XPCLR*. In the second case, it is also important to test the presence or absence of selection in each sampled deme.

### Detection of selective sweeps from mixed samples

An additional situation often encountered in natural populations is “cryptic substructure”, where a substructured population is assumed panmictic, and thus samples are taken from different demes and mixed together [65], [66]. To determine how this affects the detection of positive selection, we analyzed mixed samples, i.e. a sample selected randomly from both demes, in two scenarios: *m*20*L* and *m*0.2*G*. Both scenarios involve a strong distinction between demes, the former in selective strength and the latter in low migration rate (thus high differentiation).

**Fig 8:**
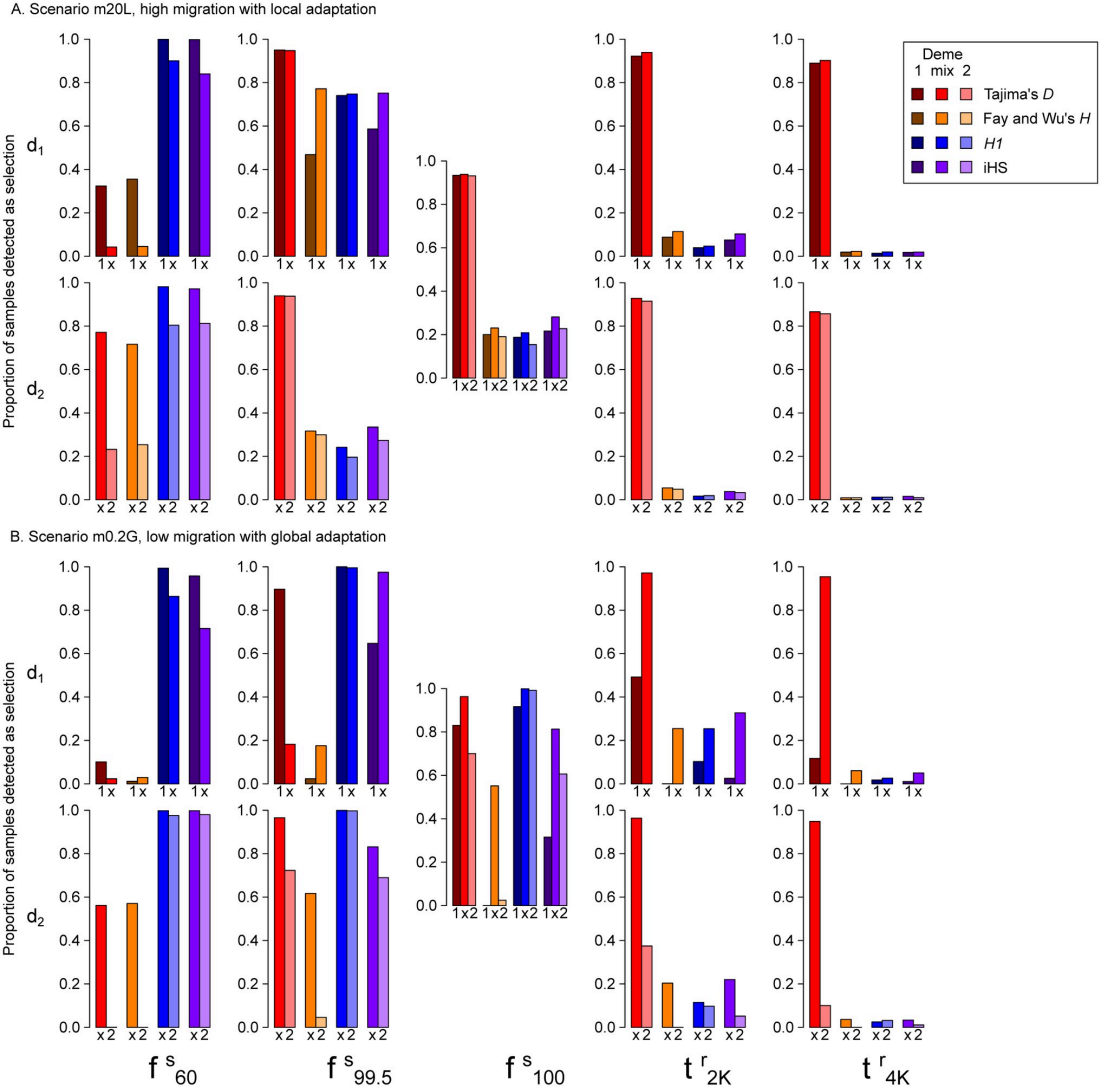
Full-locus simulation: Comparing summary statistic detection rate of sweeps between deme-specific samples and mixed samples. The proportion of samples detected as sweeps from *d*_1_, mixed samples and *d*_2_ (noted below the bars as “1”, “x” and “2”) in various time stages, for scenarios A: m20L, B: m0.2G. Different hues indicate different methods, and the shades represent *d*_1_, mixed and *d*_2_ from dark to light. $f_{100}^{s}$ (global fixation) is shared by both demes, thus the graph in the middle contains results from *d*_1_, mixed and *d*_2_. The other four time points are deme-specific, thus we must compare only one deme with mixed data.

**Fig 9:**
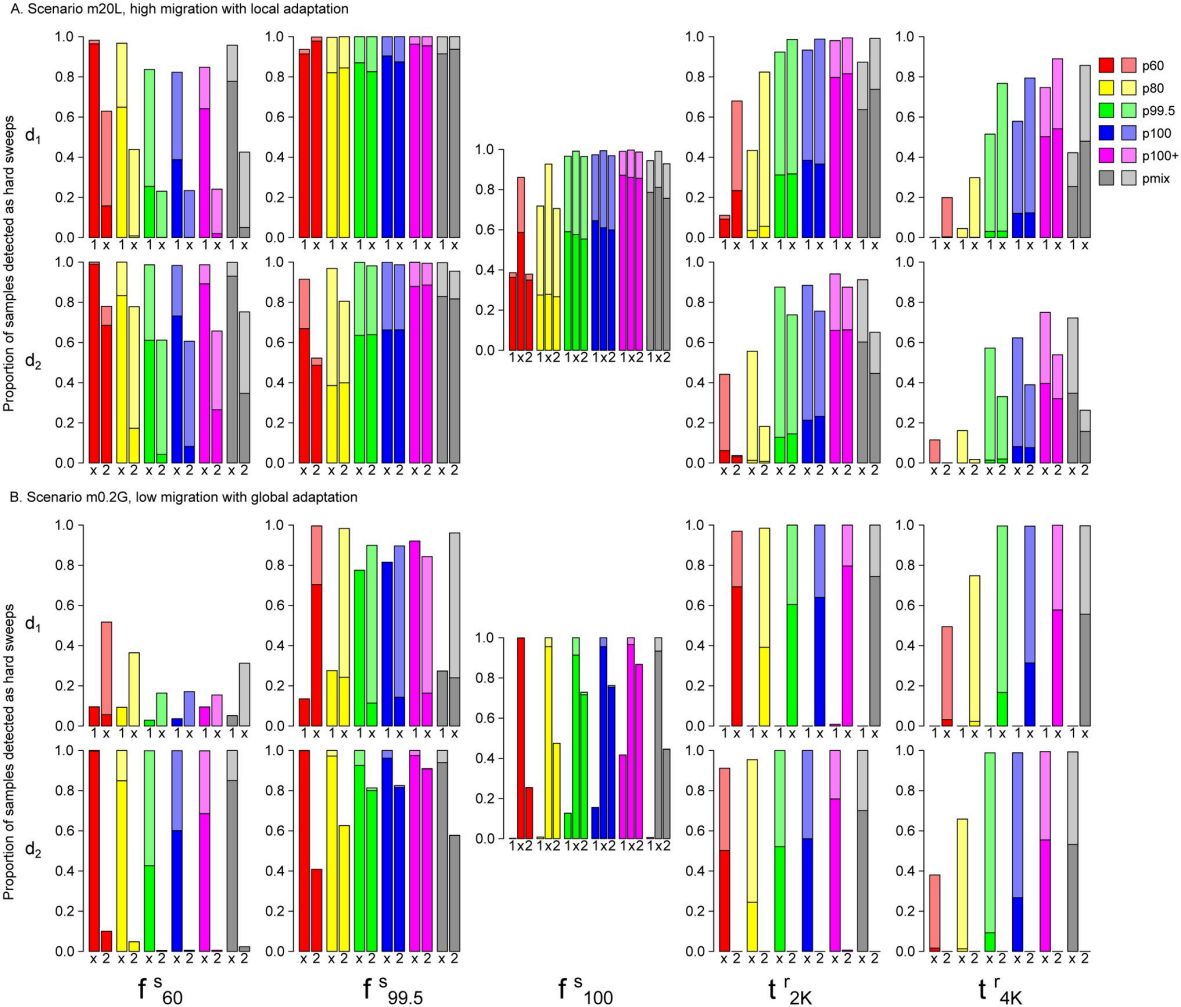
Full-locus simulation: Comparing evolBoosting detection rate of sweeps between deme-specific samples and mixed samples. The proportion of samples detected as hard (darker shade) or soft sweeps (lighter shade) by six EvolBoosting predictors, from *d*_1_, mixed samples and *d*_2_ (noted below the bars as “1”, “x” and “2”). Scenarios are A: m20L, B: m0.2G. $f_{100}^{s}$ (global fixation) is shared by both demes, thus the graph in the middle contains results from *d*_1_, mixed and *d*_2_. The other four time points are deme-specific, thus we must compare only one deme with mixed data.

In *m*20*L*, mixed samples performed generally similar to deme-specific samples (Fig 8A). For ongoing sweeps, all methods have higher power in *d*_1_ than mixed samples, and higher in mixed samples than *d*_2_. This is consistent with the fact that the adaptive allele frequency is higher in *d*_1_ than *d*_2_, and between them in mixed samples. For completed sweeps, summary statistics perform almost identically between the two demes and mixed samples; EvolBoosting, on the other hand, detects more sweeps in mixed samples than in samples taken separately from either deme (Fig 9A). However, the additional power by EvolBoosting manifests almost entirely as misclassification into soft sweeps. In other words, mixed samples intensify the “spatial softening” effect in local adaptation scenarios.

The results from *m*0.2*G* showed a different picture. Across most time stages and methods, mixed samples yielded far better detection power then *both* demes (Fig 8B). The major exception is haplotype-based methods (*H1*, *iHS*) during earlier stages, consistent with that they are less affected by population substructure. In the most extreme cases, EvolBoosting cannot detect any completed sweeps (at $t_{2K}^{r}$ and later) from separate demes, but have a close to 100% power (hard + soft combined) from mixed samples (Fig 9B). The proportion of sweeps misclassified as soft follows the same pattern of “temporal softening” as in panmictic datasets.

Further examination of the threshold values for *m*0.2*G* mixed and non-mixed samples explained the stark contrast of the detection powers. Thresholds for detecting positive selection are derived from quantiles of the neutral distribution of a statistic under the same demographic model, to control for false positive rates. For frequency-spectrum-based summary statistics and EvolBoosting predictors, the thresholds are far more stringent in separate-deme datasets in *m*0.2*G* than panmictic ones, while those for mixed samples are less stringent than panmictic ones. In other words, neutral loci from mixed samples have a distribution of allele frequencies that make sweep loci “standing out” more clearly relative to neutral loci from separate-deme samples. The reason is two-fold: First, separate-deme samples have an excess of rare alleles (migrant haplotypes) and a negative Tajima’s *D*, while mixed samples have an excess of intermediate-frequency alleles (because the deepest split in the coalescence tree represents deme split) and a positive Tajima’s *D*. Sweeps introduce more rare alleles so the contrast with the latter is stronger. Second, mixed samples have a smaller variation among neutral loci than separate-deme samples. In separate-deme samples, the number of external migrant haplotypes in each locus can be highly variable; the site frequency spectra are vastly different between a locus with zero migrant haplotypes and a locus with one. On the other hand, mixed samples always consist of two types of haplotypes which—on average—makes the site frequency spectra more homogeneous among loci.

This implies a warning that, in some cases, it is *not* a good idea to study demes separately when it comes to selective sweeps, especially under scenarios where demes exchange migrants occasionally. On the flip side, it also means that detailed demarcation of populations is not necessary for such analysis, and looking for selection signatures from the entire sample (without worrying about cryptic structure) does not generally pose a problem. Of course, the caveat of temporal and spatial softening still holds; in particular, if a soft sweep is detected, it could be because the allele is under selection only in a part of the population (our case of *m*20*L*).

### Genetic differentiation between demes during and after selective sweeps

**Fig 10:**
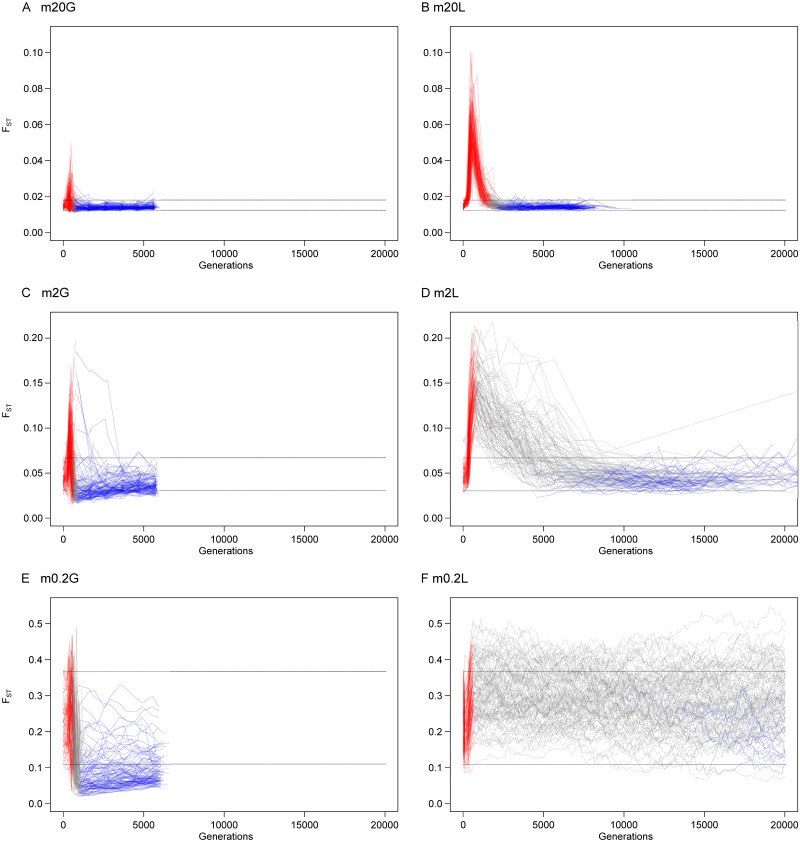
Full-locus simulation: *F*_*ST*_. The change of between-deme *F*_*ST*_ during a selective sweep and recovery. Red indicates the period before the adaptive allele reaches 99.5% in *d*_1_, blue indicates the period after global fixation, and gray for the period in-between. Each line is one replicate population. The scenarios are: A. *m*20*G*; B. *m*20*L*; C. *m*2*G*; D. *m*2*L*; E. *m*0.2*G*; F. *m*0.2*L*. For *m*0.2*L* the time points are fixed number of generations (every 100 generations) instead of based on allele frequency. Horizontal dash lines denote the 95% range of the neutral baseline *F*_*ST*_, i.e. the value at 0 generations.

Fig 10 shows how *F*_*ST*_, a inter-deme differentiation measure, changes during a selective sweep and during the recovery phase afterwards. *F*_*ST*_ is calculated as the mean value for all SNPs in the first 200kb and further averaged from 50 samples in each replicate. Here we considered six different scenarios: high (*Nm* = 20), intermediate (*Nm* = 2) and low (*Nm* = 0.2) migration, each including global and local adaptation. In all scenarios, within the first 1,000 generations, the adaptive allele reaches fixation or near-fixation in its native deme *d*_1_, resulting in an increase of *F*_*ST*_. The exact trajectories during the increase and afterwards, however, differs qualitatively between global and local selection scenarios.

With global adaptation, while generally *F*_*ST*_ reaches the peak at 400–500 generations, considerable variation exists between replicates; in a large fraction of them, *F*_*ST*_ remained at the baseline level. In particular, for low migration (*m*0.2*G*), the high baseline variance almost obscured the *F*_*ST*_ peak. As the adaptive allele takes over *d*_2_, *F*_*ST*_ quickly reduces and with lower migration even dips *below* the initial values. Even after 5,000 generations of recovery, under *m*0.2*G*, it has not returned to the baseline. In other words, the homogenizing effect of global selection lasts longer with lower migration, and persists even as most sweep-detection methods have lost their power (see previous sections). In theory, for deme pairs with low migration, exceedingly low *F*_*ST*_ values can be a signal of global selection.

Local adaptation at both high (*m*20*L*) and intermediate (*m*2*L*) migration, however, see a uniform increase of *F*_*ST*_ during the selection phase of *d*_1_. The peak is higher compared to the global adaptation counterpart, and every replicate reaches a higher value of *F*_*ST*_ than the baseline. With low migration (*m*0.2*L*), the *F*_*ST*_ values sometimes rose above the baseline but do not form a discernible peak. The return to baseline is much slower as *d*_2_ only fixes the allele by migration without selection, and the speed of returning is inversely proportional to migration rate. Note that the *F*_*ST*_ value does *not* go below the baseline. For *m*0.2*L*, the variance between replicates are so large that distinguishing different phases based on *F*_*ST*_ is impossible.

In conclusion, the relationship between selection and *F*_*ST*_ is complicated and cannot be simply described as “increased” or “decreased” differentiation. *F*_*ST*_ or other differentiation measures have been extensively used as a proxy for selection detection [67], [68], [69]; in particular, a higher *F*_*ST*_ than the genomic baseline is considered a signature of local adaptation. A metastudy has suggested that the high neutral variation of *F*_*ST*_ baseline can cause false positive of local adaptation [70], which can occur when migration rate is low. Our results suggests that the efficacy of *F*_*ST*_ outliers (higher than baseline) as a marker of local adaptation depends qualitatively on the migration rate, and is particularly useful for intermediate to high migration rates (*Nm* >= 2). Higher migration leads to short detection window as *F*_*ST*_ recovers quickly when the adaptive allele “diffuses” to other demes, while lower migration causes the neutral variation to be too high to produce a meaningful contrast.

We also would like to emphasize that while *F*_*ST*_—selection links are mostly discussed in the context of local adaptation, it can also prove useful for *global* adaptation. In this case, it is a reduced *F*_*ST*_ value, particularly when the baseline is high (lower migration), being a signature of adaptation. However, note that purifying selection can also decrease *F*_*ST*_ values of sites directly affected [4], and adaptive sites tend to occur around conserved sites because they are both in “functional” regions like coding or promoter sequences. On the other hand, background selection slightly increases *F*_*ST*_ of surrounding neutral sites by reducing local *N*_*e*_; the effects are complicated but should not be as quantitatively prominent as those incurred by selective sweeps. It remains to be tested, therefore, how much would adaptation-caused *F*_*ST*_ reduction “stand out” in an already functional (negatively selected) region. In addition, purifying selection does not reduce haplotype diversity in contrast to sweeps [71], thus a round of screening with haplotype-based methods (such as *iHS*) can alleviate this problem.

### Conclusion

We have established the concepts of temporal misclassification and spatial softening. Temporal misclassification, including softening and hardening, refers to classification of hard sweeps as soft or vice versa, because the training model mismatches with the tested data in time stage. Spatial softening can cause hard sweeps in neighboring demes to be falsely detected as soft. In addition, if a panmictic population model is used in data analysis but the real situation involves occasional migration, false positive sweeps (mainly classified as soft) may ensue. All three processes can produce signals of soft sweep without an actual soft sweep present. Therefore, the claim that human populations have overwhelmingly soft sweeps as the mode of adaptation may be a result of biased classification. To have a more complete view of hard and soft sweep frequencies in human and other populations, one needs to take into account migration as a demographic process (instead of only population size change), and explicitly distinguish different stages of selective sweeps.

Under lower migration, especially when the migration rate between demes is at or below the order of 1-2 migrants per generation, panmictic assumptions break down and extra care must be exercised. This affects frequency-spectrum-based methods more than the haplotype-based ones. Thus only the latter can be reliable in low migration cases to detect ongoing or recent sweeps. The effects of occasional migrants must be taken into account when searching for selective sweeps. Inter-deme differentiation statistics, in particular *F*_*ST*_, make it difficult to distinguish global and local selection in early stages, but they have better sensitivity for local selection under higher than lower migration. Our results can guide future studies on populations in patchy habitats, such as island and desert populations, for example, *Tillandsia landbeckii* in the Atacama Desert (Merklinger et al. Under Review).

## Supporting information

S1 File The single-site simulation algorithm with documentation. The Perl script used for simulating single-site adaptation allele frequency trajectory, under two-deme and three-deme scenarios.
(PL) Click here for additional data file.

S2 File The full-locus simulation algorithm with documentation. The R script used for simulating 600kb genomic regions under a selective sweep, including all custom-defined functions and the main script.
(R) Click here for additional data file.

S3 File Simulations with the program SLiM, with a higher number of replicates and a larger sample size. The methods and results from simulating *m*0*G* and *m*20*L* scenarios with SLiM, and analyzing the data with the same methods as in the main text.
(DOCX)

S1 Fig Single-site simulation: Population size and time. A, C, E: The time taken for an adaptive allele from the beginning (mutation event) to reach a frequency of 99.5%. B, D, F: The duration of selection phase length, defined as the time between the adaptive allele reaching 5% and 99.5%. The migration rates are 0.02 (A, B), 2 (C, D) or 200 (E, F).
(PDF)

S2 Fig Results from evoNet, a deep learning algorithm for detecting and classifying selective sweeps. The proportion of samples classified as hard (darker colors) or soft (lighter colors) by three evoNet classifiers trained with sweeps in different time stages. A: Panmictic populations. B-I: Subdivided populations, including four scenarios each containing two demes.
(PDF)

S3 Fig Full-locus simulation: The detection rate of various methods in various scenario, zoomed in version for pre-fixation time stages. The proportion of samples detected as selective sweeps by various methods, under the scenarios: A-C. *m*0*G*, D-F. *m*20*Ld*_1_, G-I. *m*20*Ld*_2_, J-L. *m*0.2*G*. Only the time stages at or before global fixation are shown; the horizontal axis denotes adaptive allele frequency instead of generations. The vertical line indicates time of 100% fixation. A,D,G,J. Power of seven summary statistics; dashed lines indicate haplotype-based methods. B,E,H,K. Proportion detected by six EvolBoosting predictors *correctly* as hard sweeps. C,F,I,L. Proportion detected by six EvolBoosting predictors *incorrectly* as soft sweeps. The general qualitative pattern is similar to *d*_1_.
(PDF)

S4 Fig Full-locus simulation: The detection rate of various methods in global adaptation scenarios in the non-native deme. The proportion of samples detected as selective sweeps by various methods, under the scenarios: A-C. *m*20*G*, D-F. *m*2*G*, G-I. *m*0.2*G*, in *d*_2_ where the adaptive allele is imported to. The vertical line indicates time of 100% fixation. A,D,G. Power of seven summary statistics; dashed lines indicate haplotype-based methods. B,E,H. Proportion detected by six EvolBoosting predictors *correctly* as hard sweeps. C,F,I. Proportion detected by six EvolBoosting predictors *incorrectly* as soft sweeps. The general qualitative pattern is similar to *d*_1_.
(PDF)

S1 Table Summary of single-site simulations. For all two-deme and three-deme scenarios, the parameters, mean and standard deviation of key time points and time lengths. “Waiting Period” is the length of time from beginning of simulation to the *last* generation where *d*_1_ and *d*_2_ do *not* contain any adaptive allele.
(XLSX)

S2 Table Selective sweep detection rate. The proportion of all samples detected as selected sweeps by all our detection methods. Here “2p” indicates a demographically correct neutral control, while “1p” indicate control with panmictic neutral samples. “200k” indicates only the first 200kb of data is used for analysis, and “600k” indicates that the entire region is used.
(XLSX)

S3 Table Selective sweep detection rate, comparison between different sample sizes. Comparison of detection rates by summary statistics and evolBoosting, on population data simulated by R (*n* = 50) and SLiM (*n* = 100).
(XLSX)
