# Supplementary material for: Adaptation in structured populations and fuzzy boundaries between hard and soft sweeps
Source: PLoS Comput Biol. 2019 Nov 11;15(11):e1007426. doi: 10.1371/journal.pcbi.1007426 (PMC6872172; doi:10.1371/journal.pcbi.1007426)
Supplement: S3 File — The methods and results from simulating m0G and m20L scenarios with SLiM, and analyzing the data with the same methods as in the main text. (DOCX) [file pcbi.1007426.s003.docx]

Supplementary Text

1. Methods for SLiM simulations

SLiM 3.2 (Haller 2019) is a simulation programming operated by the programming language Erdos. We used SLiM to generate a larger number of replicates than our main datasets produced by R script, and from them we derived samples with increased sample size.

The population scenarios are *exactly* the same as “m0G” and “m20L” in the main text: “m0G” is a panmictic population of N = 20,000 haploids, and “m20L” is a subdivided population with two demes each with N = 10,000 haploids, where the adaptive mutation is only selected for in the deme it originated in and neutral in the other one. 1,000 replicates are simulated in each scenario.

To determine how sample size affects our results, a sample size of 100 individuals per deme are taken instead of 50 as in the main text. From each population, 10 samples were taken, making 10,000 total samples per scenario per time stage.

Neutral background samples were generated with ms for both m0G (panmictic) and m20L (subdivided) scenarios. For m0G, 100,000 neutral samples were generated, and for m20L, 50,000 were generated. Similar to the main text, the subdivided neutral sample contains two demes that can be separated into two samples. All statistics were calculated for the neutral samples and trained classifiers were used on them, to obtain threshold values to control for false positives.

To test whether the mutation and recombination models from our R script is comparable to SLiM, we additionally simulated 500 replicates each of a “toy population”; the population size is 2,000 haploids or 1,000 diploids, and the sequence length is 200kb. Mutation and recombination rate were both increased by 10 times compared to the main full-locus simulation, to keep region total θ and ρ at the same values. These populations were initialized with ms (*ms 2000 1 -t 80 -r 80 5000 -p 10*) evolved 5,000 generations. Then we calculated three population-level statistics: θ_π_, θ_w_ and H_1_ (haplotype homozygosity), using a sliding window scheme of window size 100kb and step size of 10kb.

2. Results: Comparison of basic population statistics between R and SLiM simulations

To ensure the results from R and SLiM mutations are comparable (next section), we must first demonstrate that the populations evolved by the two methods are equivalent. We show this in two steps. Fig S3-1 is the population-level statistics for R and SLiM simulations, each 500 replicates of N_e_ = 2,000 haploids evolved 5,000 generations. As seen, both estimates of θ are identical between methods, as is H_1_ which measures haplotype structure. This indicates that the (neutral) mutation model and recombination model are both equivalent in the two simulation methods.

The second step is to show that the selection models are equivalent. In the R simulations, selection is implemented with a pseudo-diploid model, where every chromosome (haploid individual) has a fitness value based on the genotype of itself and its “partner”; in the SLiM simulations, the model is full diploid with co-dominance. As seen in Fig S3-2, for both panmictic and subdivided model, the time since the beginning (when the adaptive allele has only 1 copy) to various milestones (the allele reaching 20%, 40%, 60%, 80% or 99.5% in either deme, and final fixation) are identical between methods. In other words, the average trajectory of the adaptive allele frequency are the same, indicating the selection model including adjustments for diploidy is equivalent between the two methods.


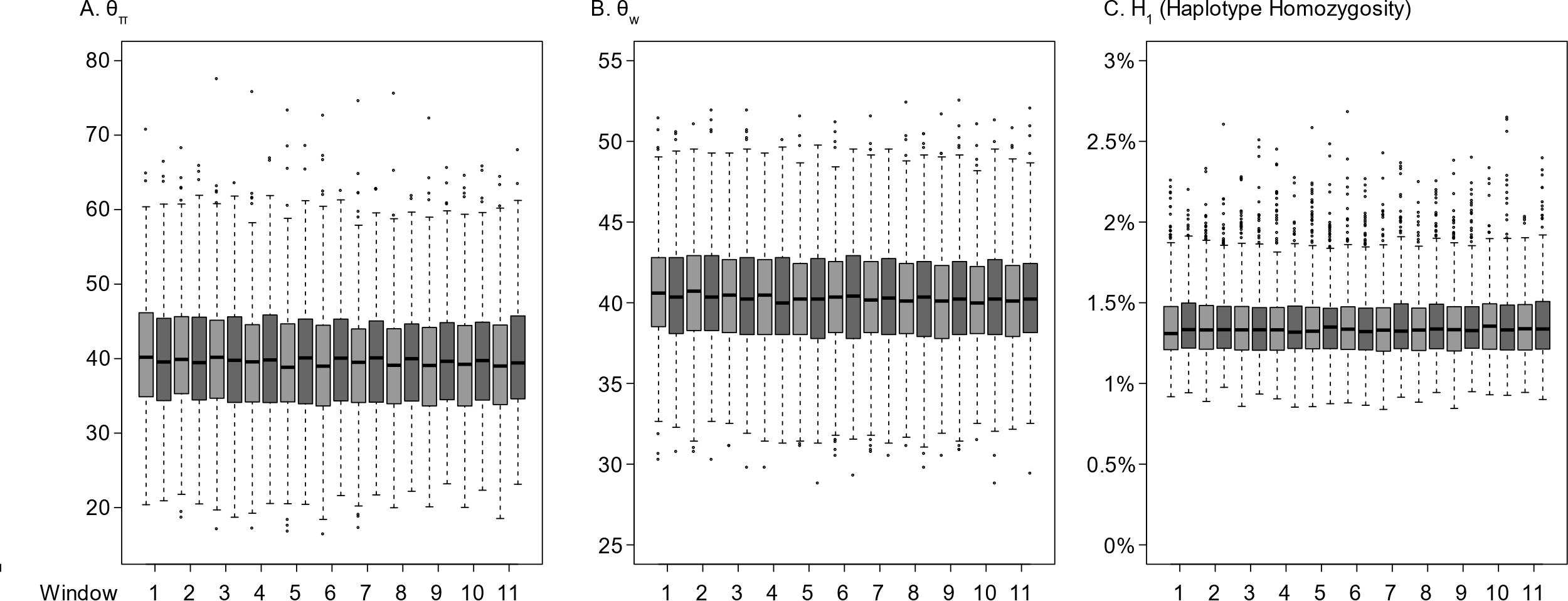


**Figure S3- 1 Population statistics in neutral simulations by custom R script (light gray) and SLiM (dark gray). The horizontal axis is the sliding window, with “1” = 0~100kb, “2” = 10~110kb, …, “11” = 100~200kb. The statistics are (A) Nucleotide diversity (an estimator of θ), (B) Watterson’s estimator of θ, and (C) Haplotype homozygosity, i.e. the probability of two randomly chosen chromosomes have the exact same sequence within the window.**


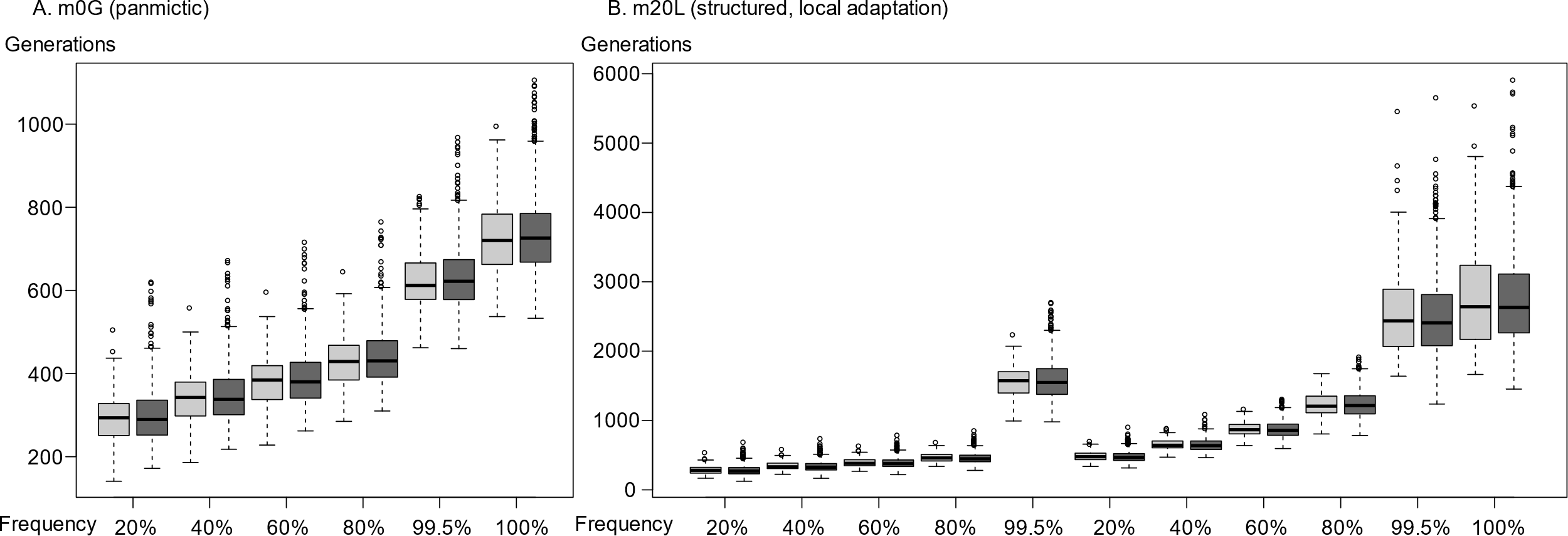


**Figure S3- 2 Comparison between simulations of selective sweeps by custom R script and SLiM; the time from beginning to milestones of adaptive allele frequency. (A) Panmictic population, scenario m0G. (B) Subdivided population with local adaptation, scenario m20L; milestone times are calculated separately for deme 1 and 2, with the 100% being the point of global fixation.**

3. Results: Detection of positive selection from SLiM simulated data

In Table S3, we listed the detection power of various methods on “old” (simulated with R) and “new” (simulated with SLiM) samples. As we demonstrated that the methods are equivalent, the differences lie only in replicate number (100 for R and 1000 for SLiM) and sample size (50 for R and 100 for SLiM). Replicate number does not affect detection rate systematically, only their precision. Therefore, the trends observed represents the change of statistics’ power based on sample size.


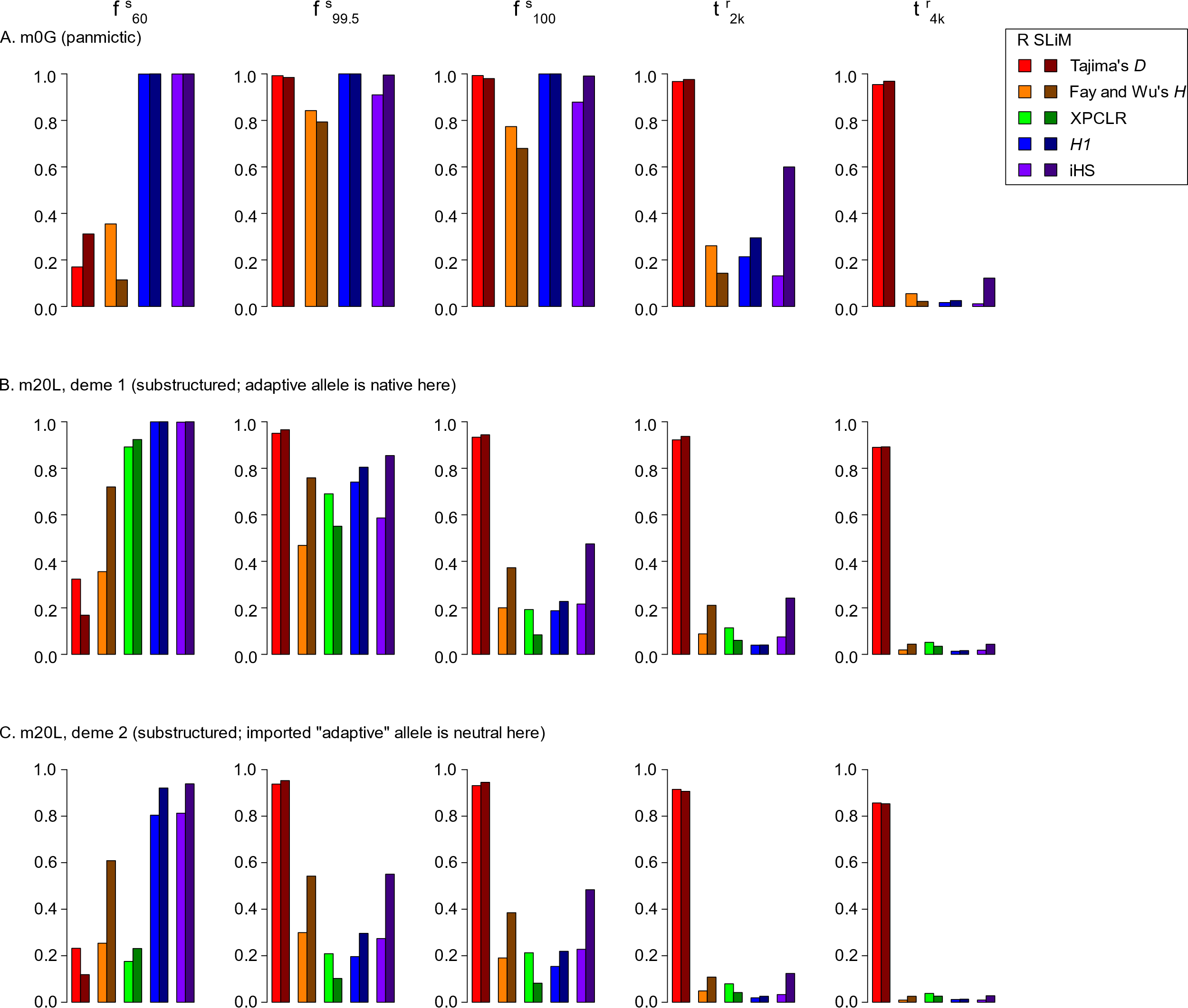


**Figure S3- 3 The power of five selected statistics used for detecting selective sweeps. Comparison between custom R script (lighter) and SLiM (darker) simulations. Five time-stages are chosen to represent ongoing, fixed and past selective sweep events.**

Figure S3-3 shows the power of five statistics: Tajima’s D, Fay & Wu’s H and XPCLR are based on allele frequency spectrum, (with XPCLR requiring comparison between demes and thus only available for m20L) and H1 and iHS based on haplotype structure. Generally, the detection powers are qualitatively similar between the two simulated datasets. While the general trend is a ~10% improvement of detection power in n=100 samples compared to n=50 samples, this difference is only consistent for haplotype-based methods. For methods using the allele frequency spectrum, the detection power for n=100 is higher in some cases (F&W’s H in m20L) and lower in others (F&W’s H in m0G, most of XPCLR).


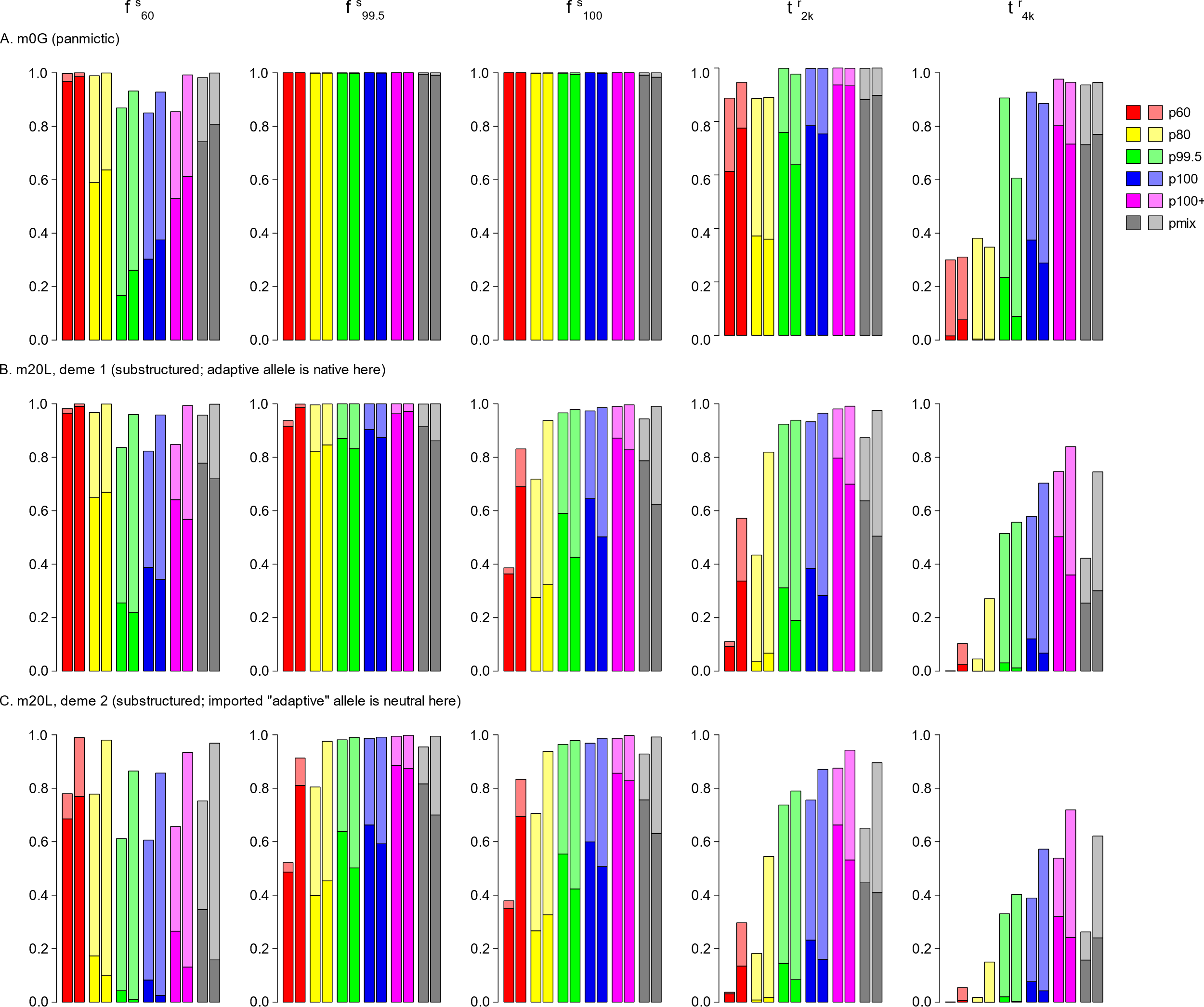


**Figure S3- 4 The power of six evolBoosting predictor pairs for detecting selective sweeps. Comparison between custom R script (left) and SLiM (right) simulations. Five time-stages are chosen to represent ongoing, fixed and past selective sweep events.**

Figure S3-4 shows the detection powers of evolBoosting at five different time-stages. With a few exceptions, SLiM datasets, with larger sample sizes, yielded higher detection rate than R datasets. This is particularly evident for p60 and both ends of the time axis. However, in many cases the increase is only in (misclassified) soft sweeps, particularly for the local adaptation scenario. In other words, temporal and spatial softening is not mitigated by increasing sample size.

Haller BC, Messer PW. SLiM 3: Forward Genetic Simulations Beyond the Wright-Fisher Model. Molecular Biology and Evolution. 2019;36(3):632-637.
