# Supplementary figures and images for: Adaptation in structured populations and fuzzy boundaries between hard and soft sweeps
Source: PLoS Comput Biol. 2019 Nov 11;15(11):e1007426. doi: 10.1371/journal.pcbi.1007426 (PMC6872172; doi:10.1371/journal.pcbi.1007426)

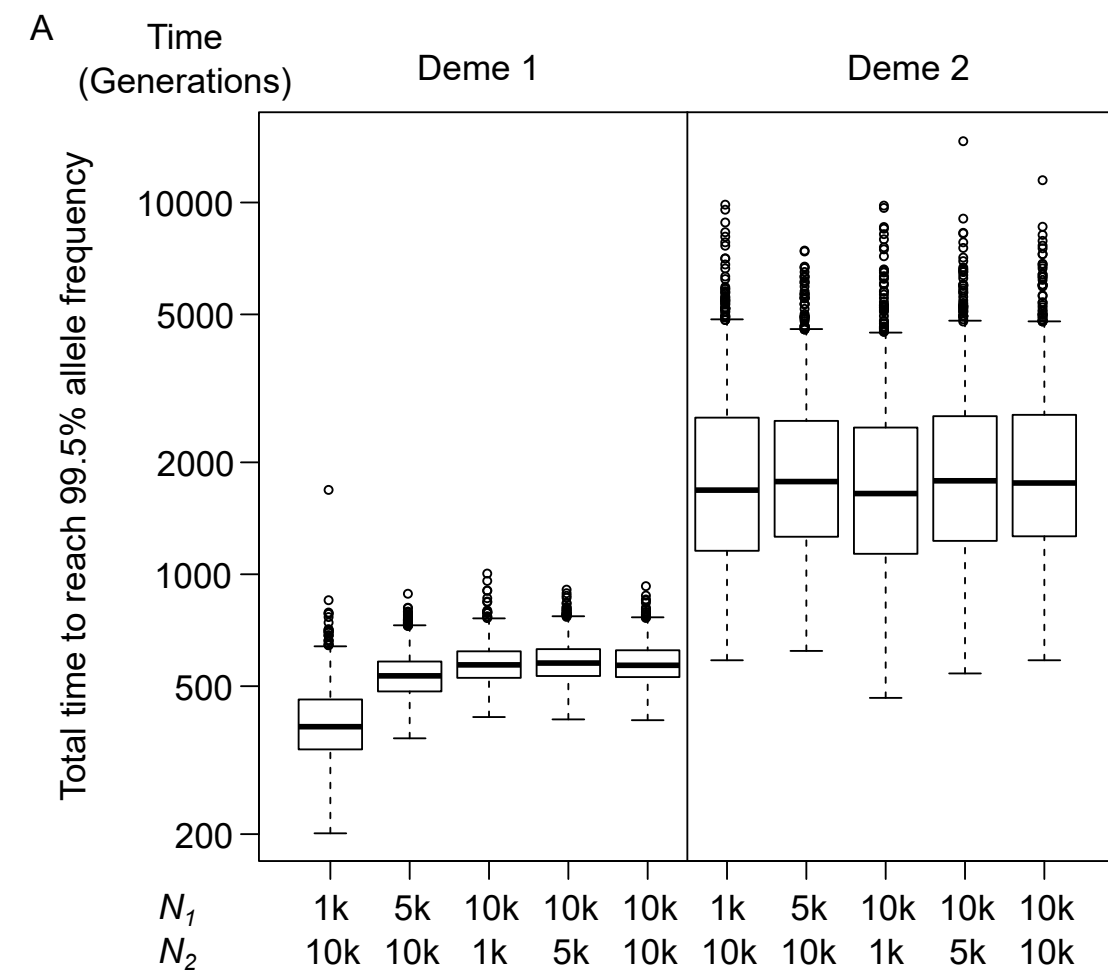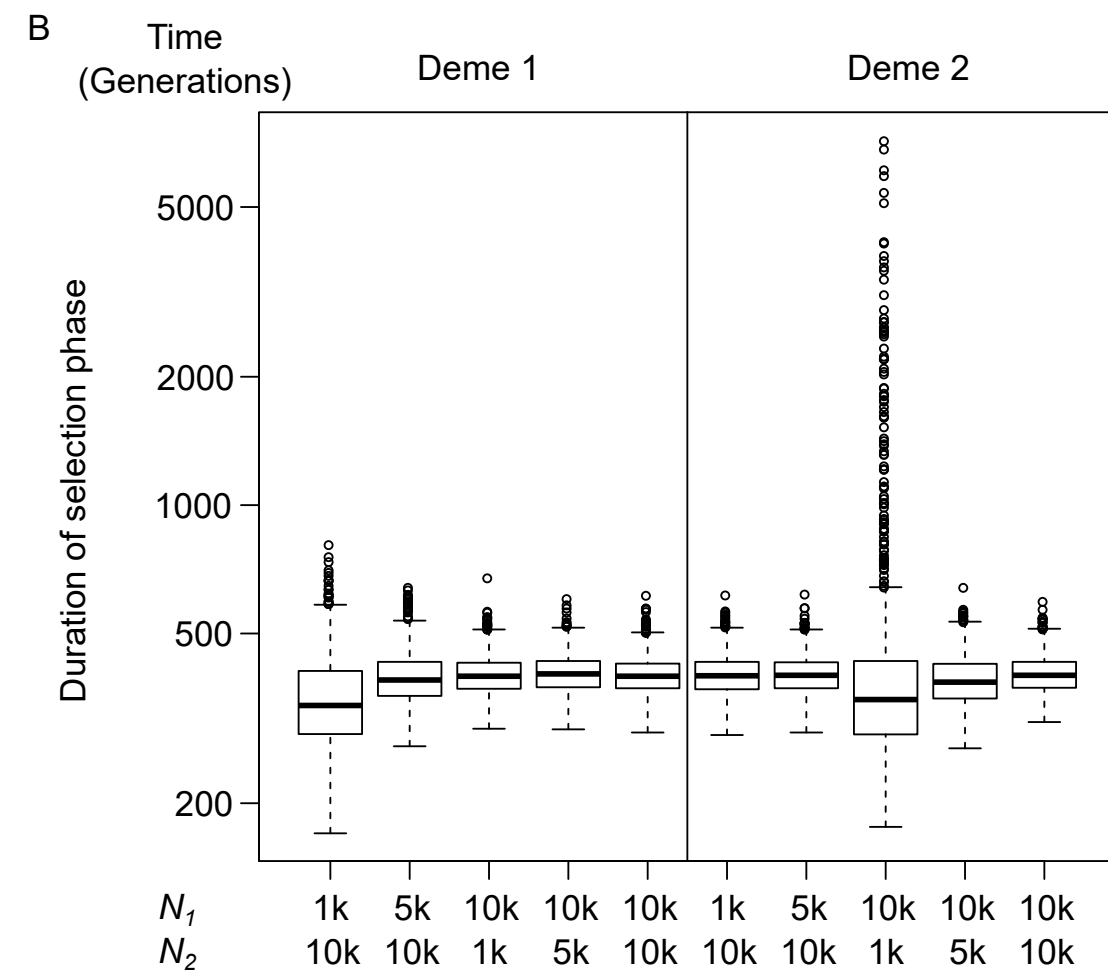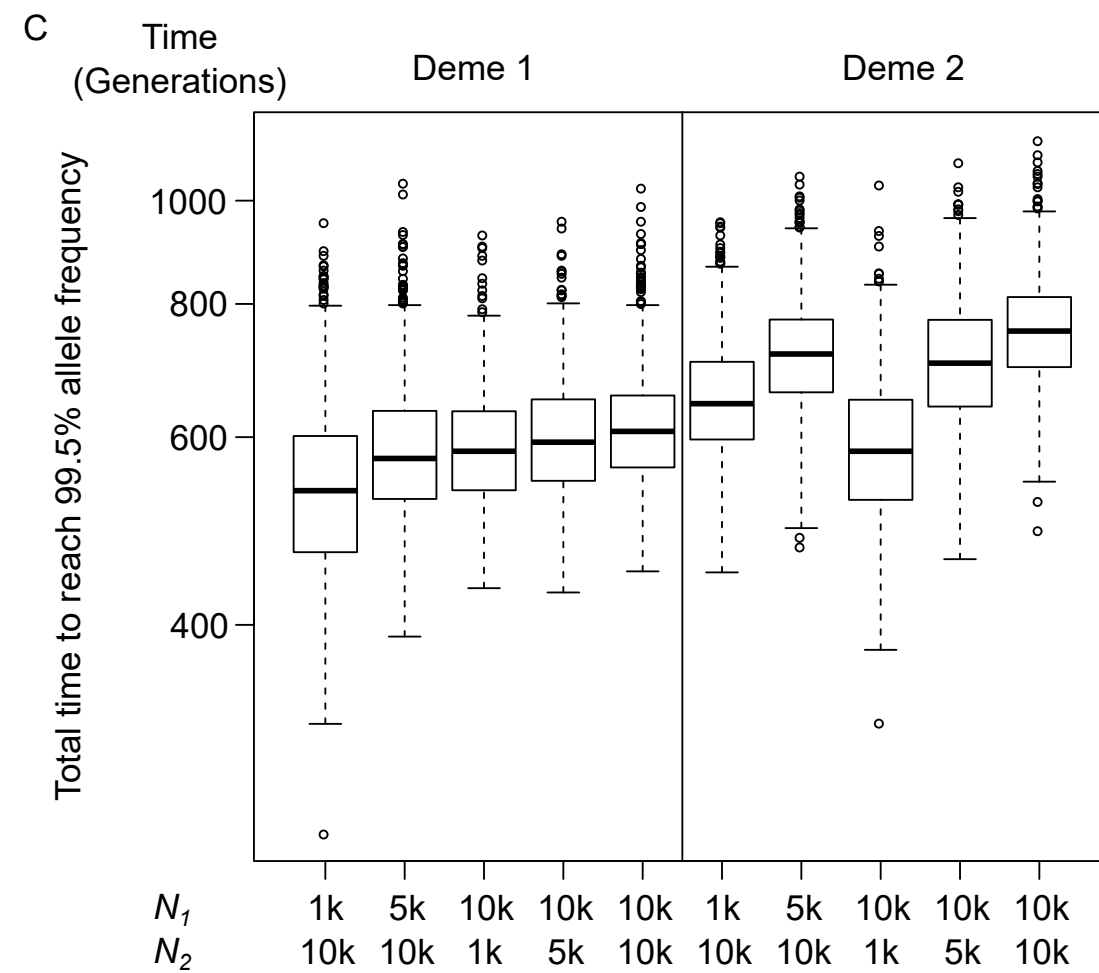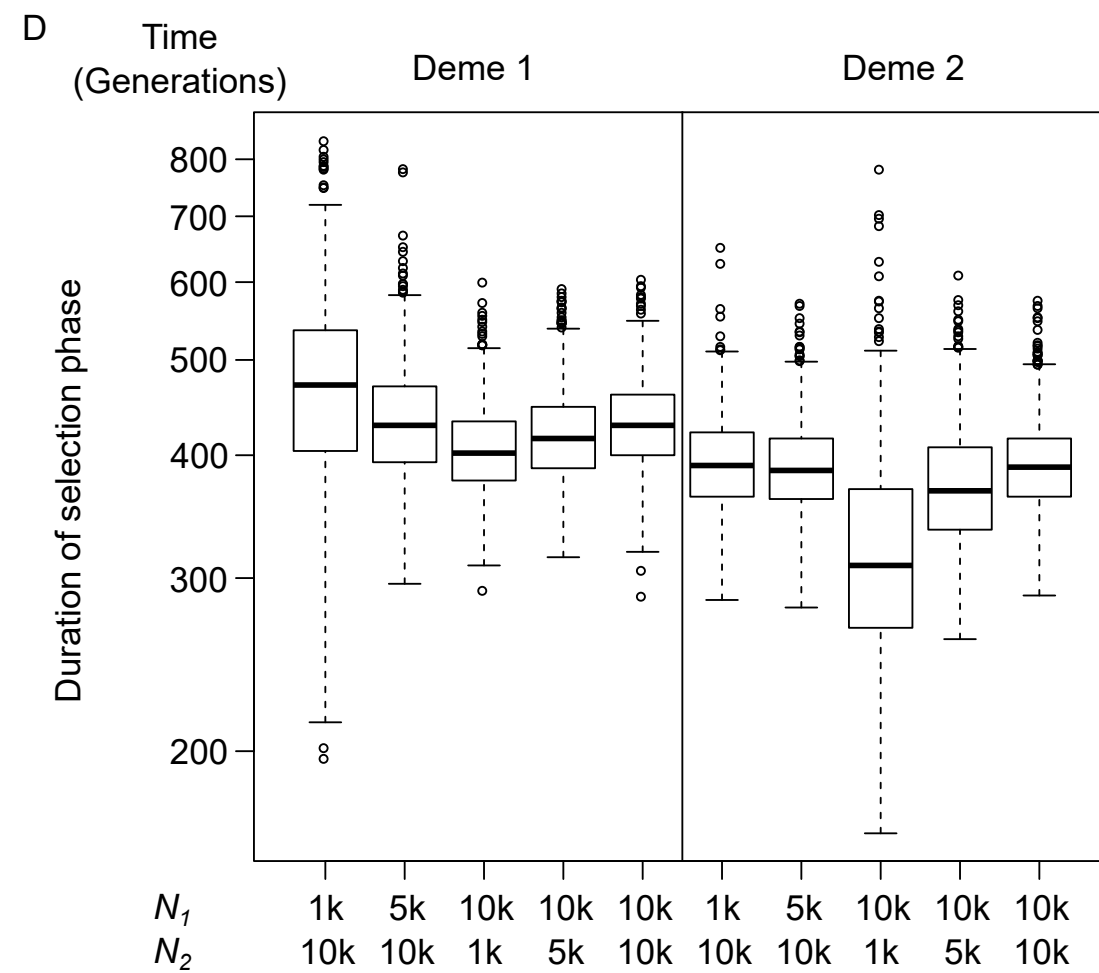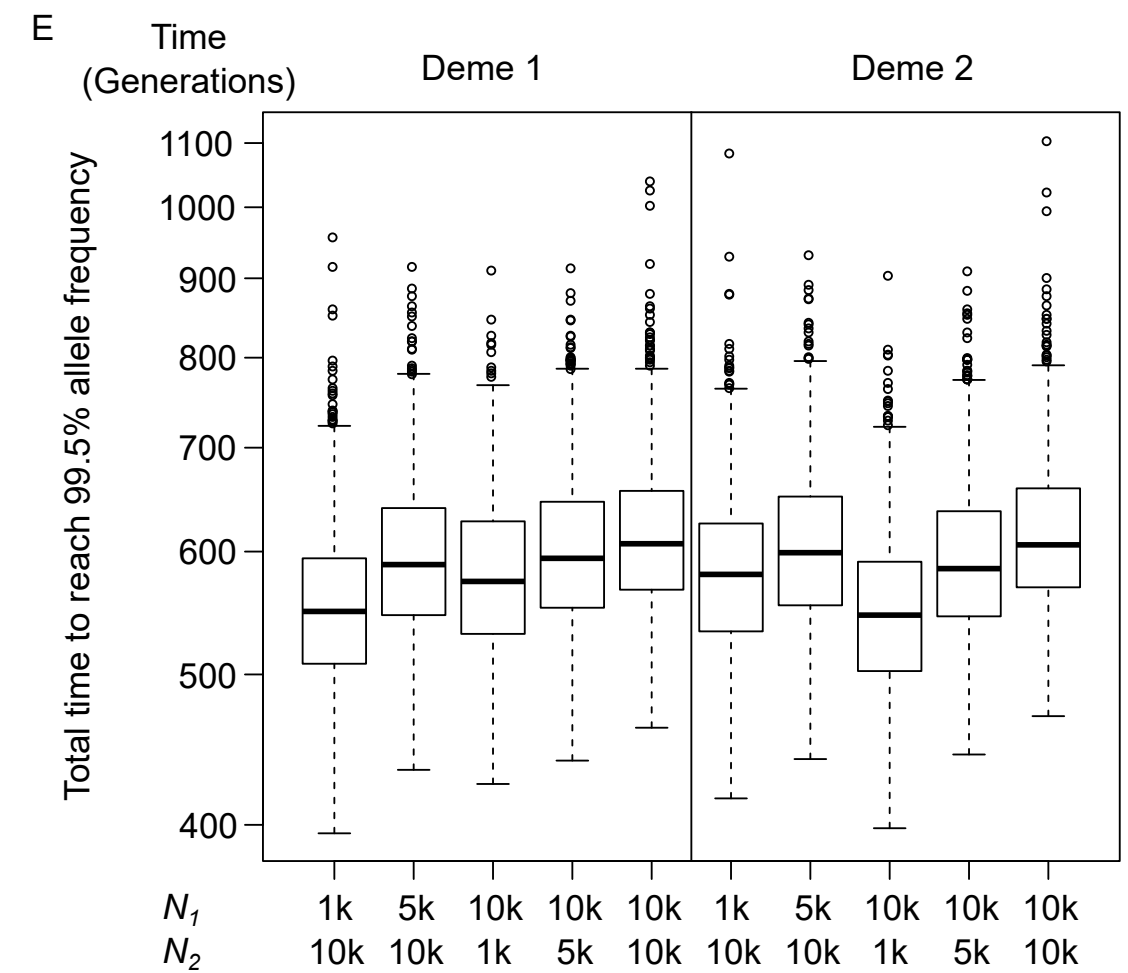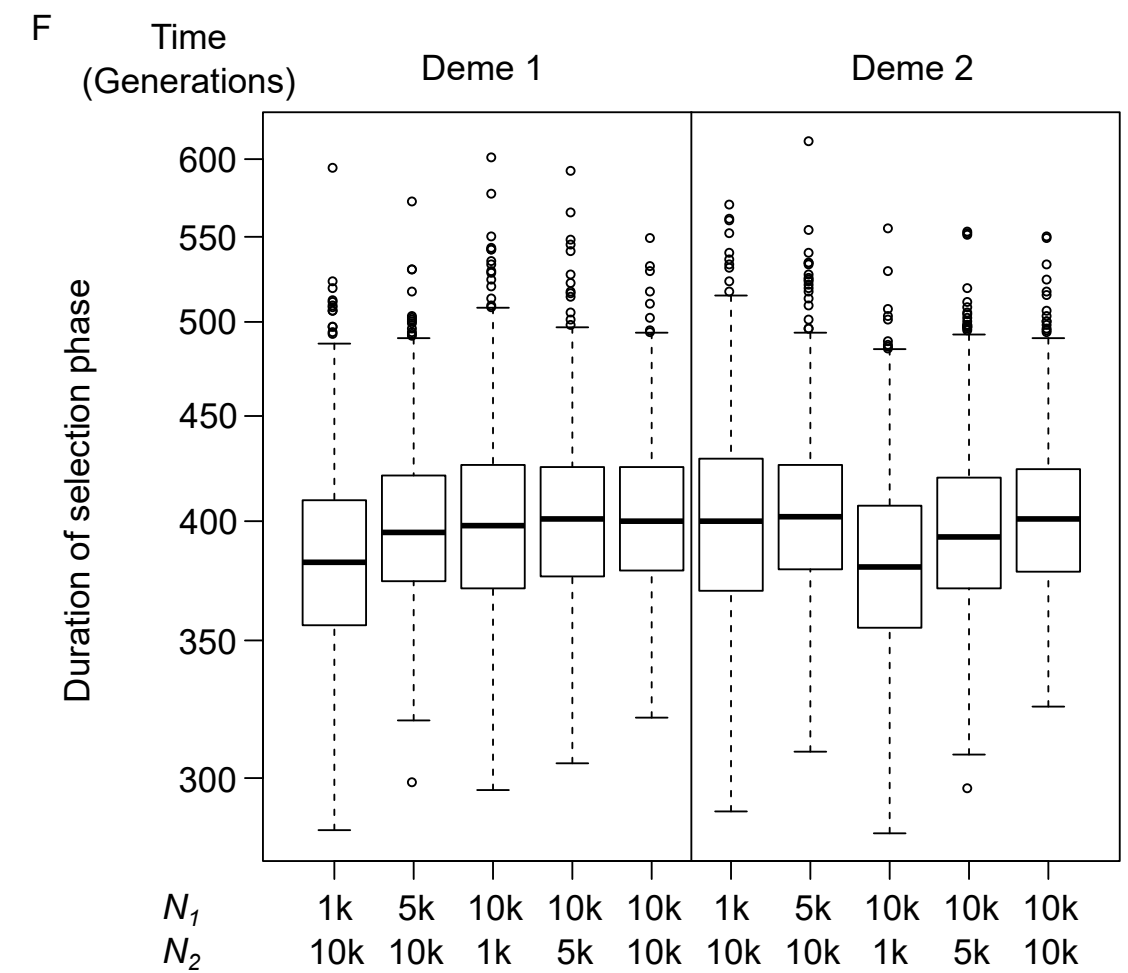

Supplement: S1 Fig — A, C, E: The time taken for an adaptive allele from the beginning (mutation event) to reach a frequency of 99.5%. B, D, F: The duration of selection phase length, defined as the time between the adaptive allele reaching 5% and 99.5%. The migration rates are 0.02 (A, B), 2 (C, D) or 200 (E, F). (PDF) [file pcbi.1007426.s004.pdf]

A m0G

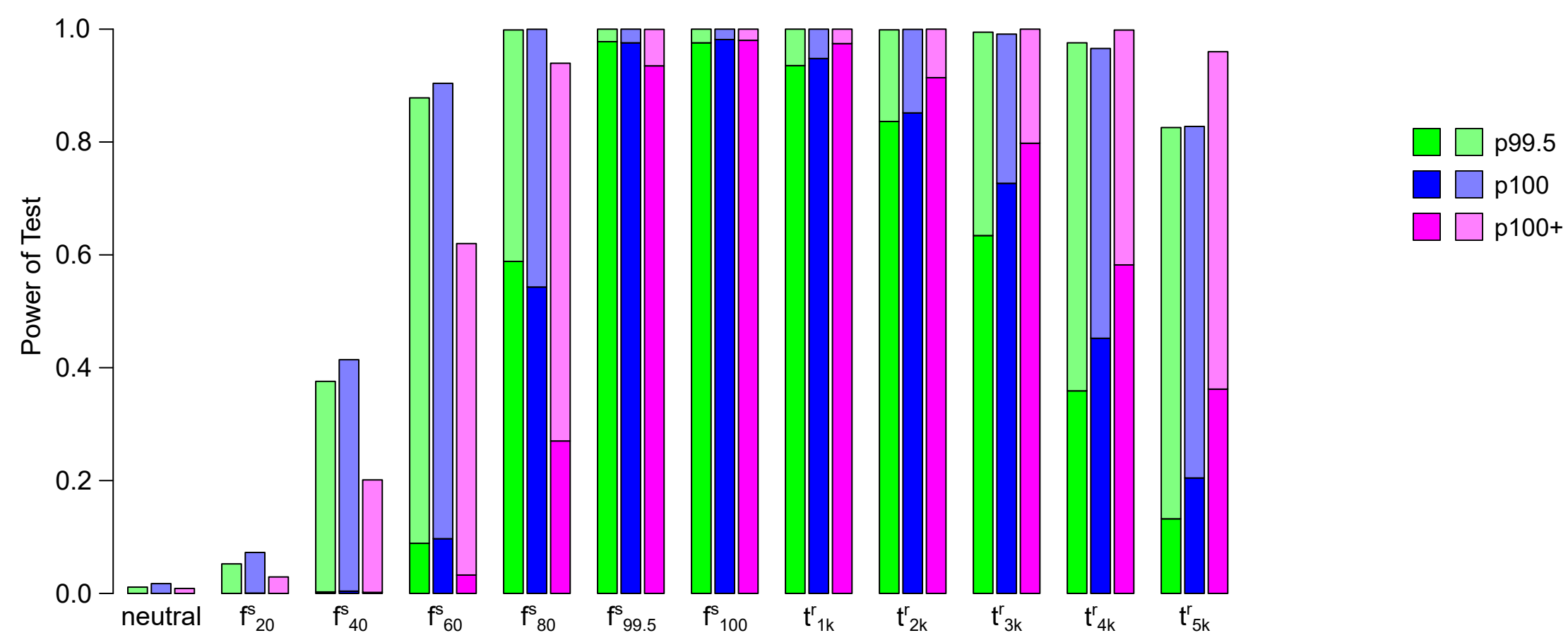

B m20G, deme 1

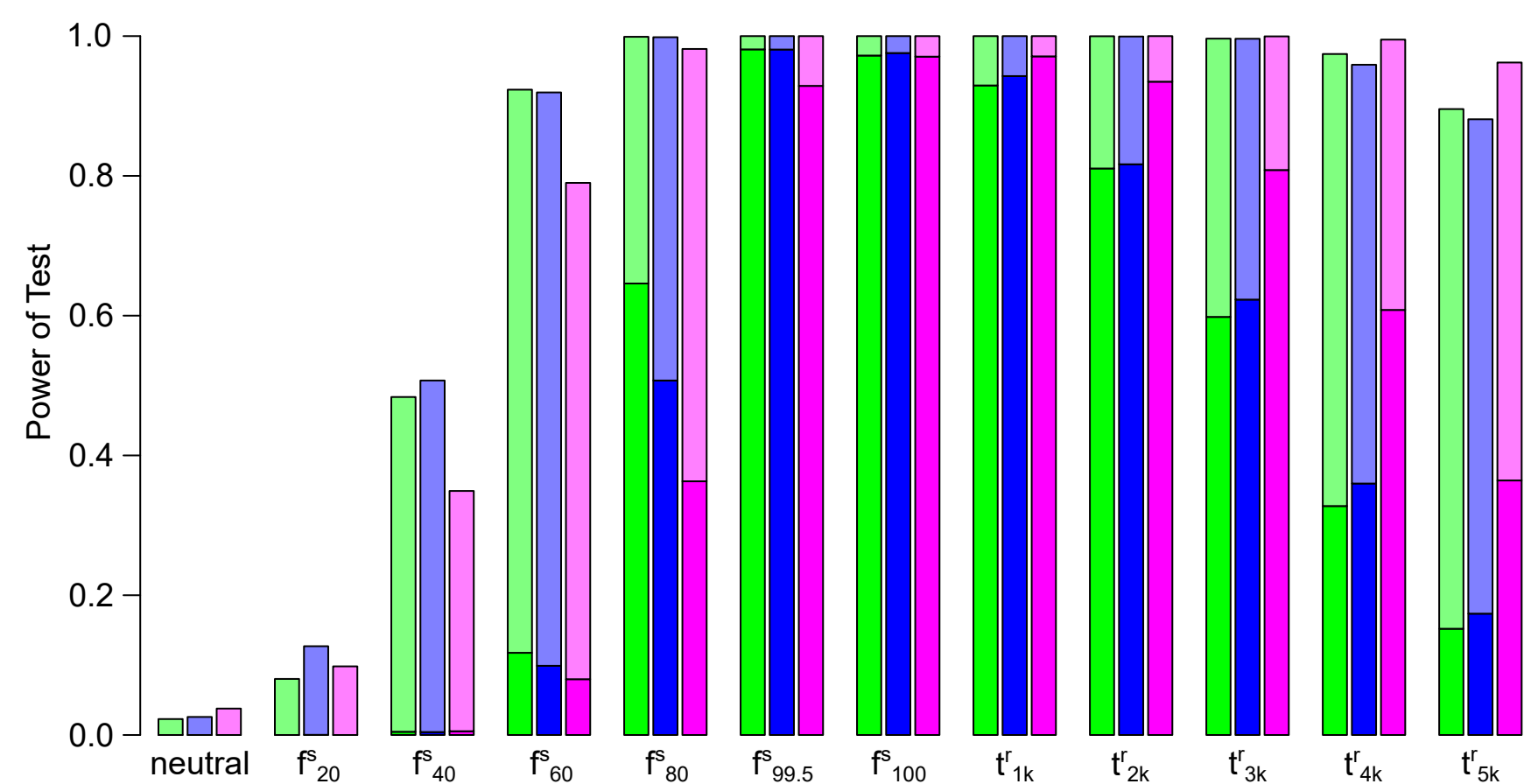

C m20G, deme 2

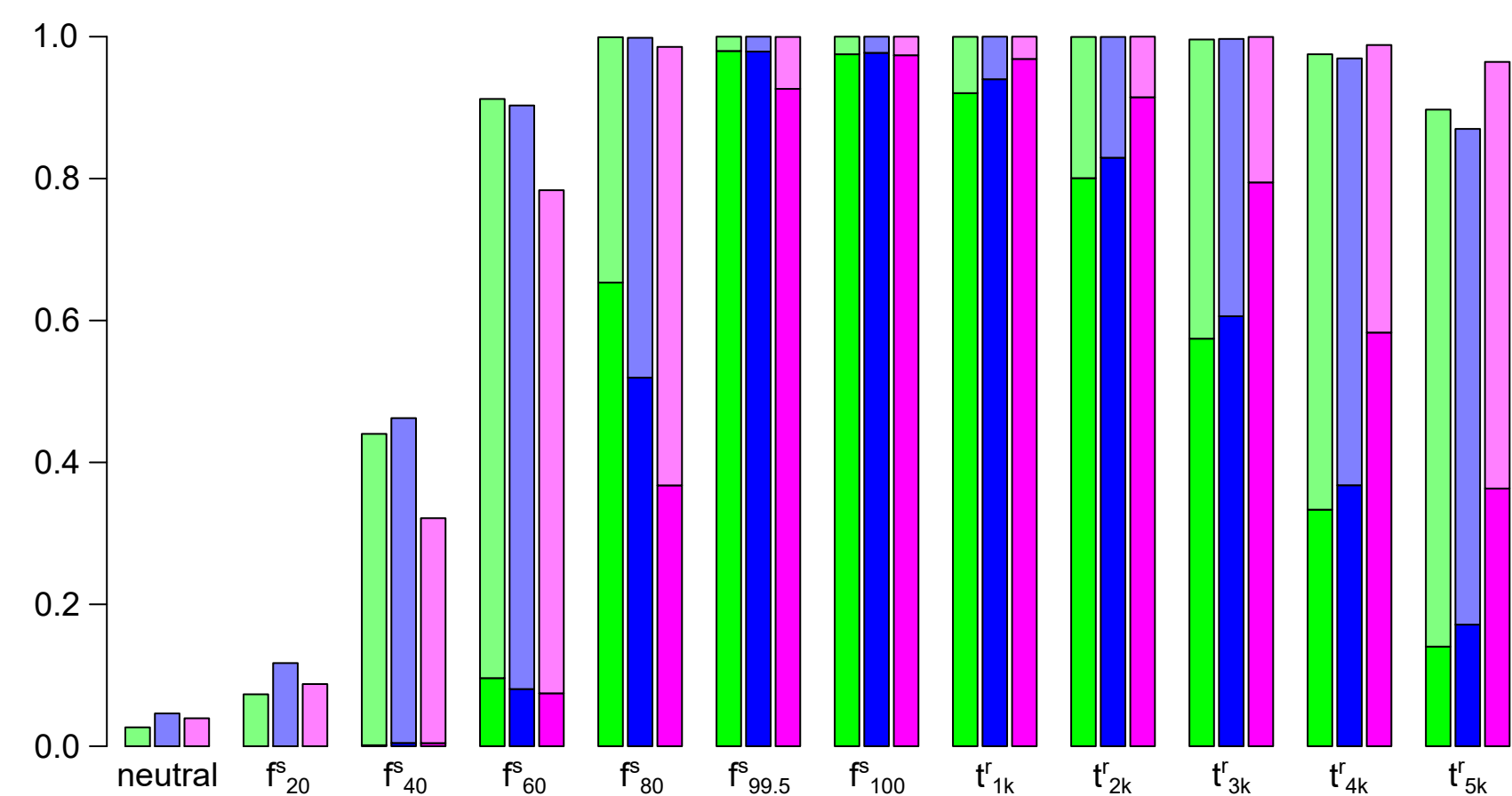

D m20L, deme 1

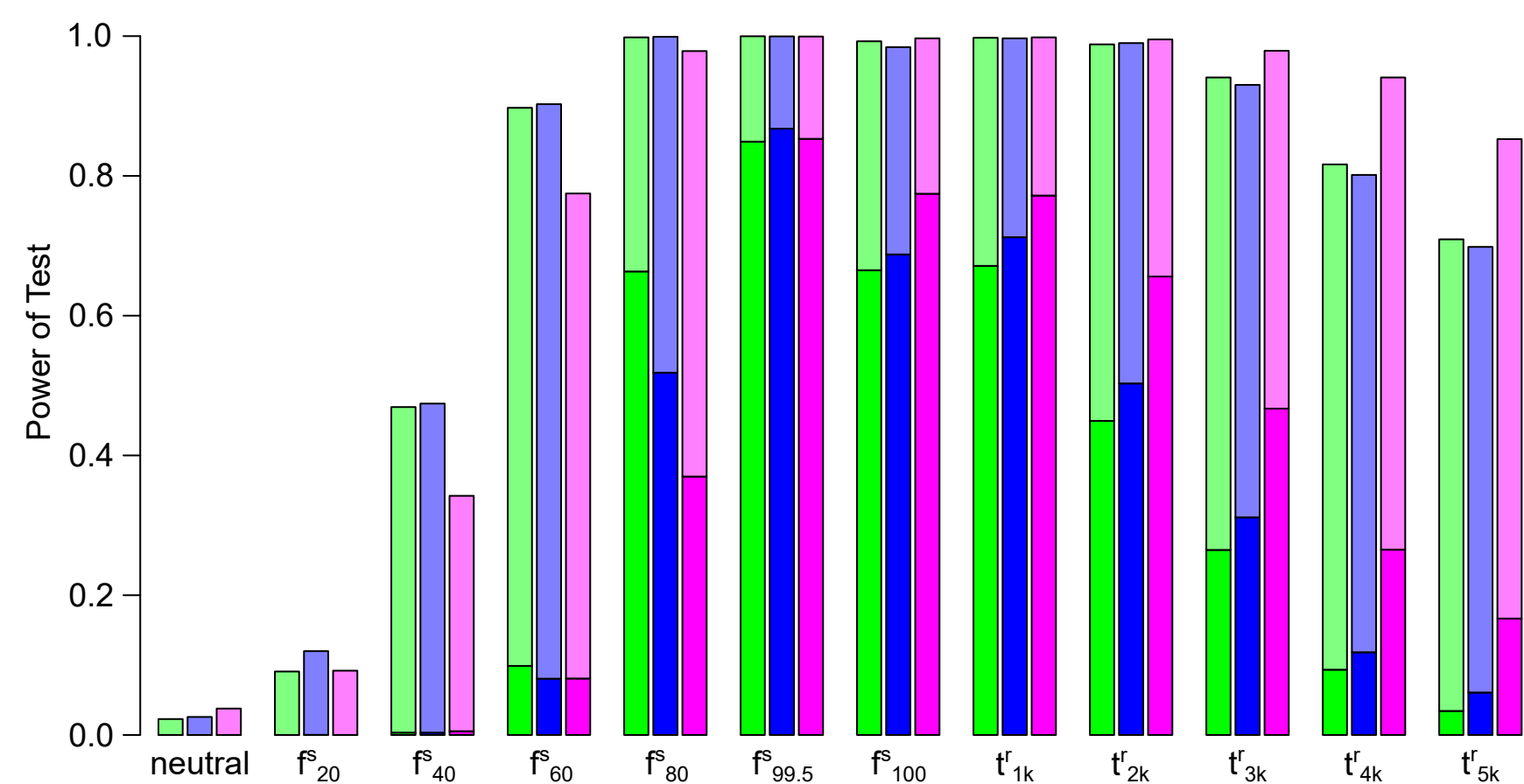

E m20L, deme 2

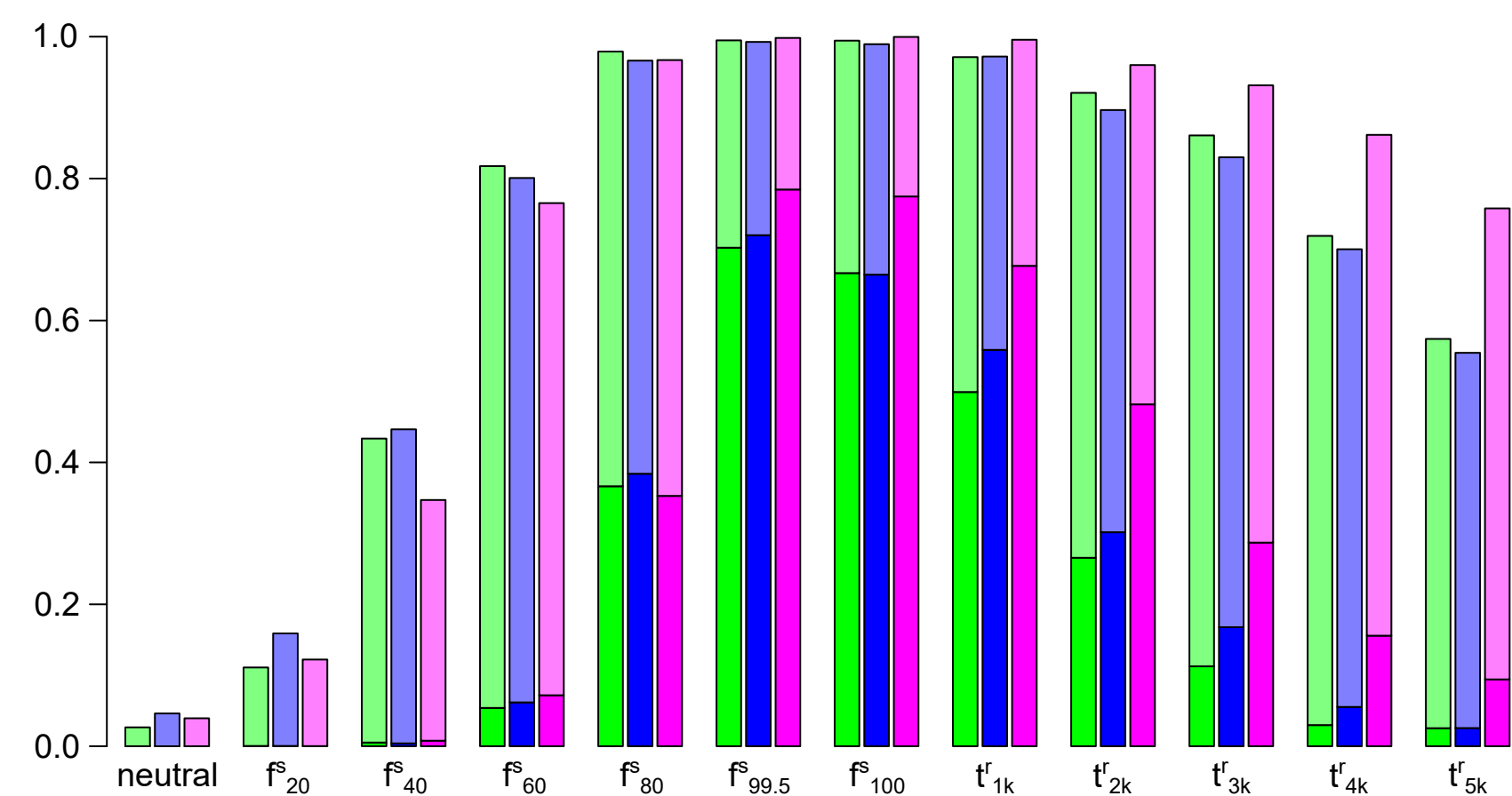

F m2G, deme 1

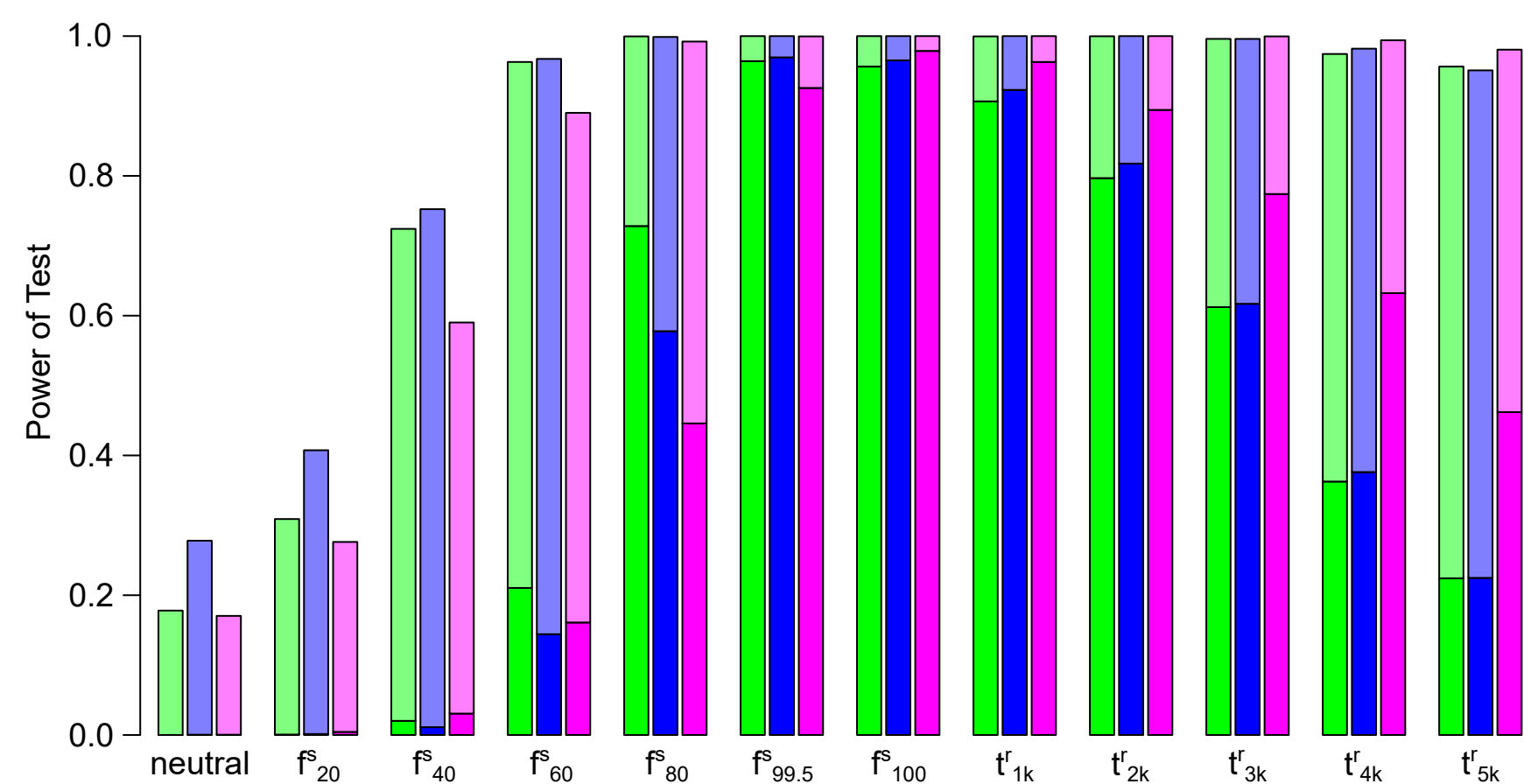

G m2G, deme 2

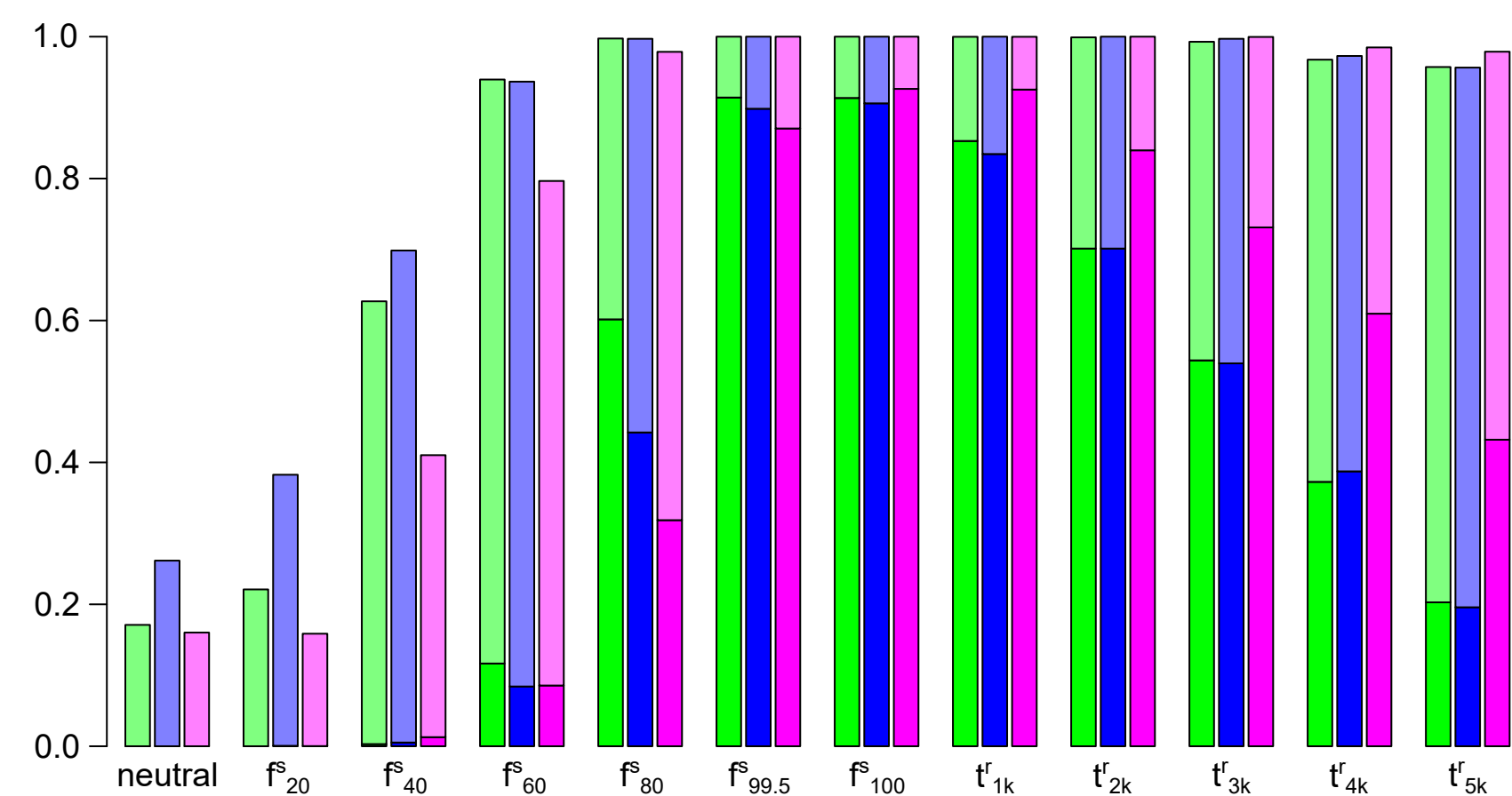

H m0.2G, deme 1

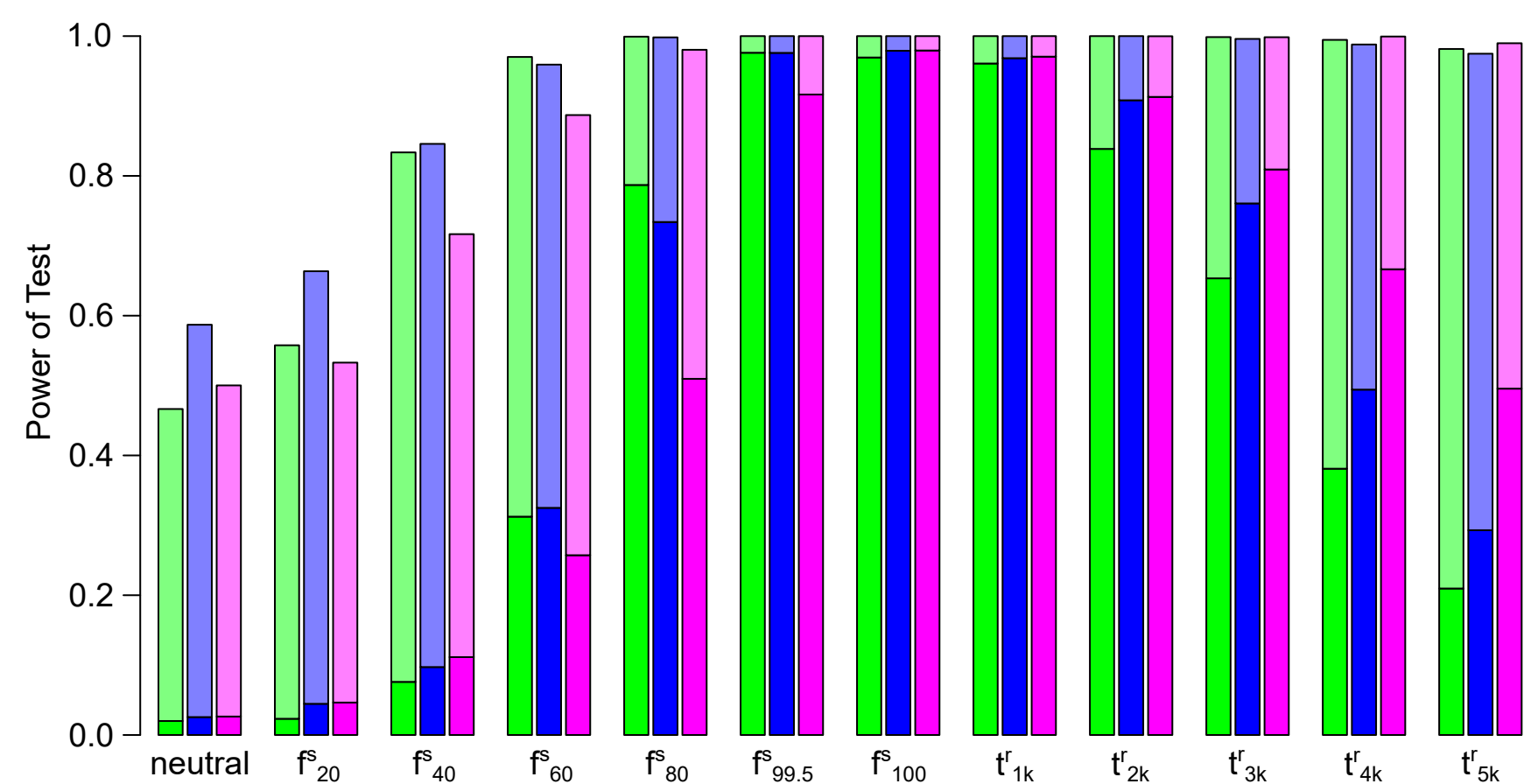

I m0.2G, deme 2

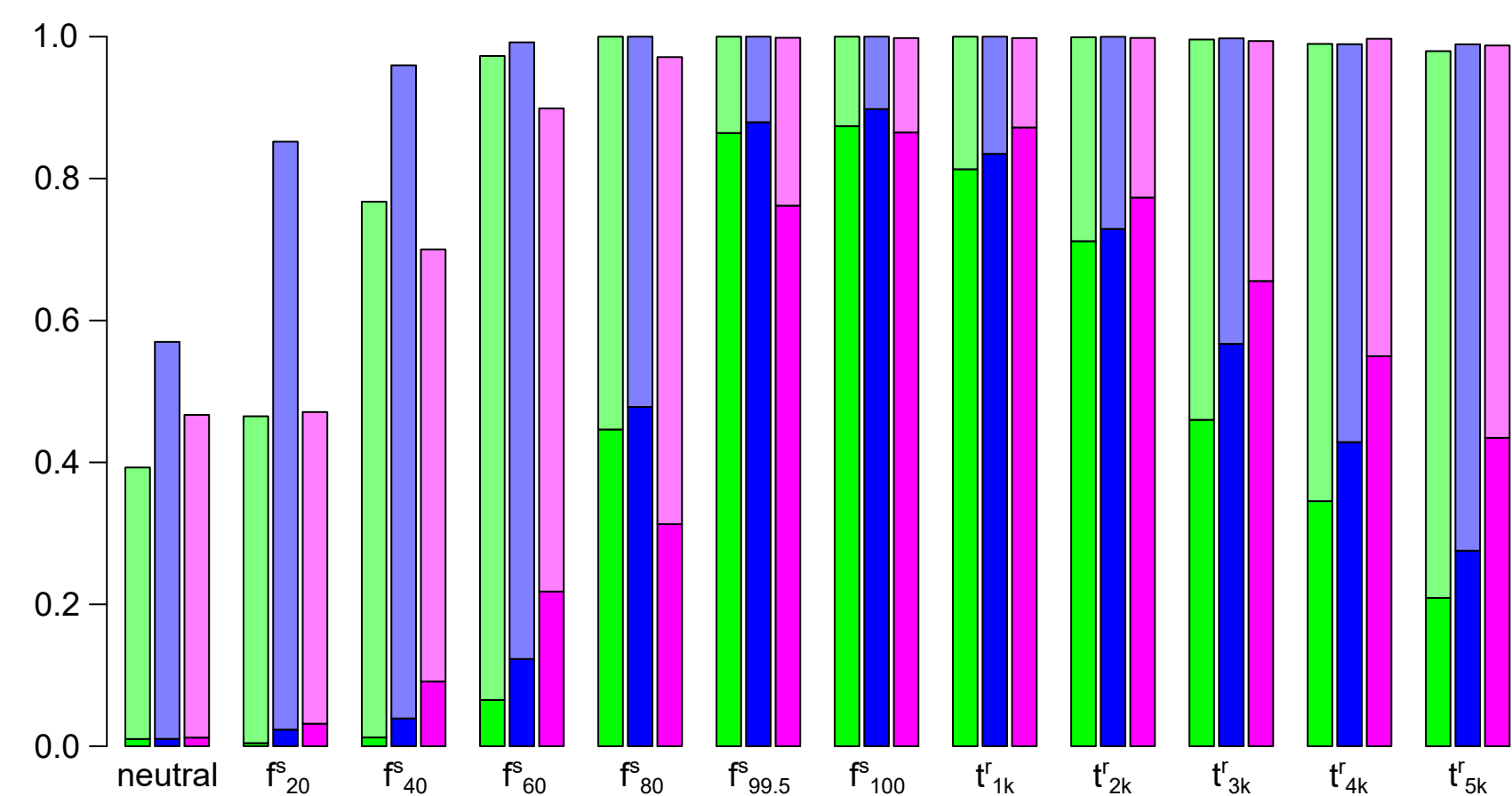

Supplement: S2 Fig — The proportion of samples classified as hard (darker colors) or soft (lighter colors) by three evoNet classifiers trained with sweeps in different time stages. A: Panmictic populations. B-I: Subdivided populations, including four scenarios each containing two demes. (PDF) [file pcbi.1007426.s005.pdf]

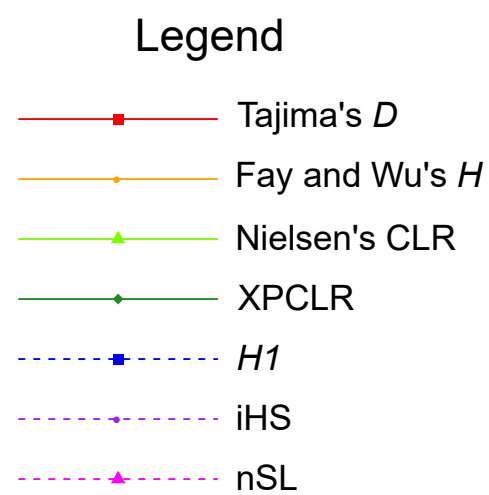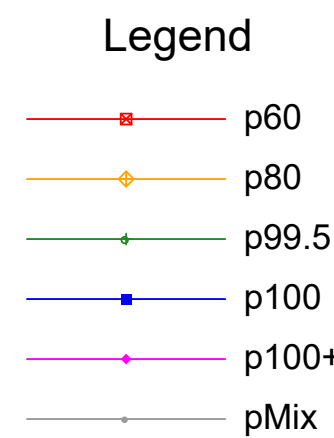

Summary Statistics

Evolboosting - Hard Sweeps

Evolboosting - Soft Sweeps

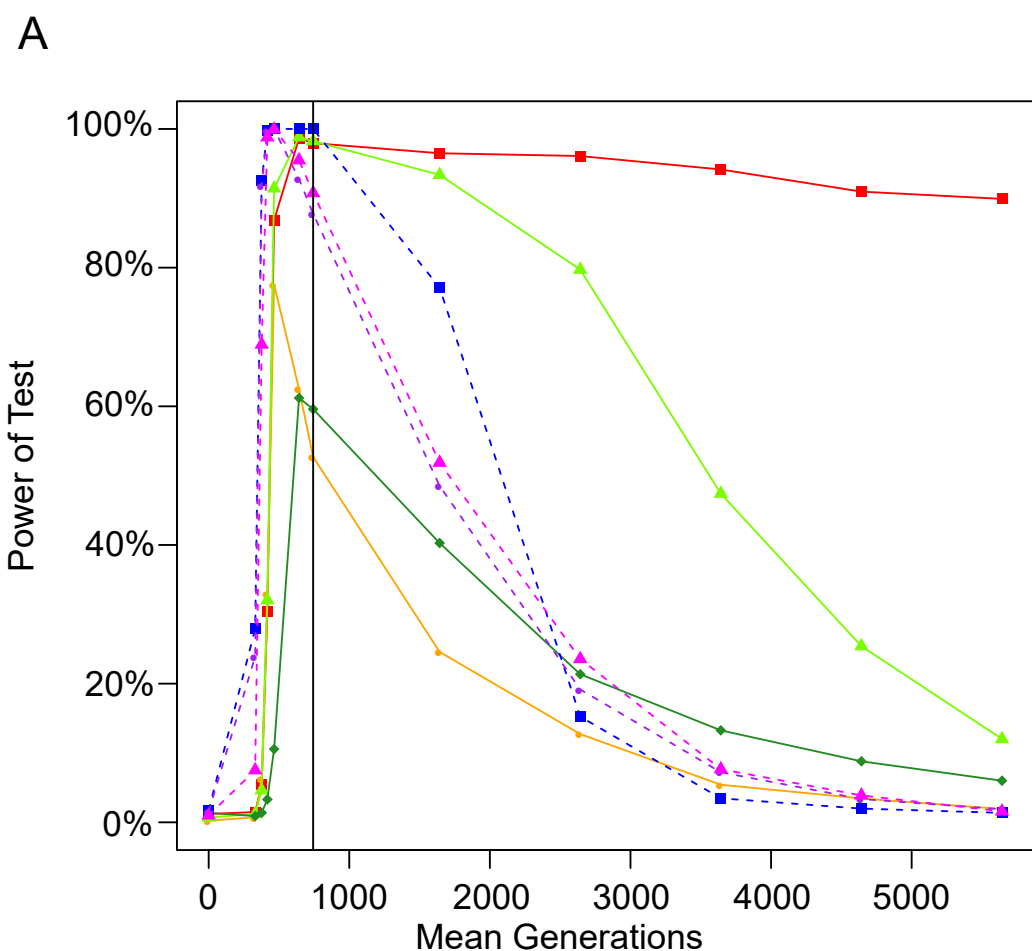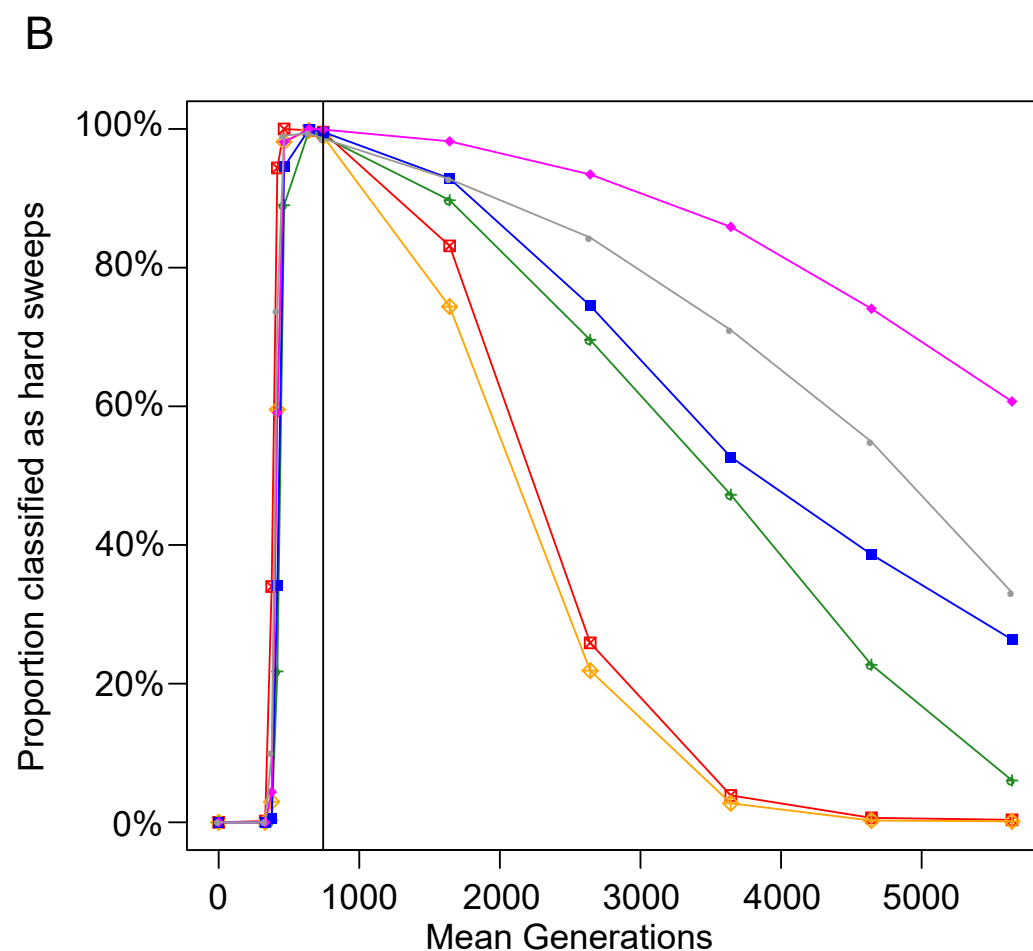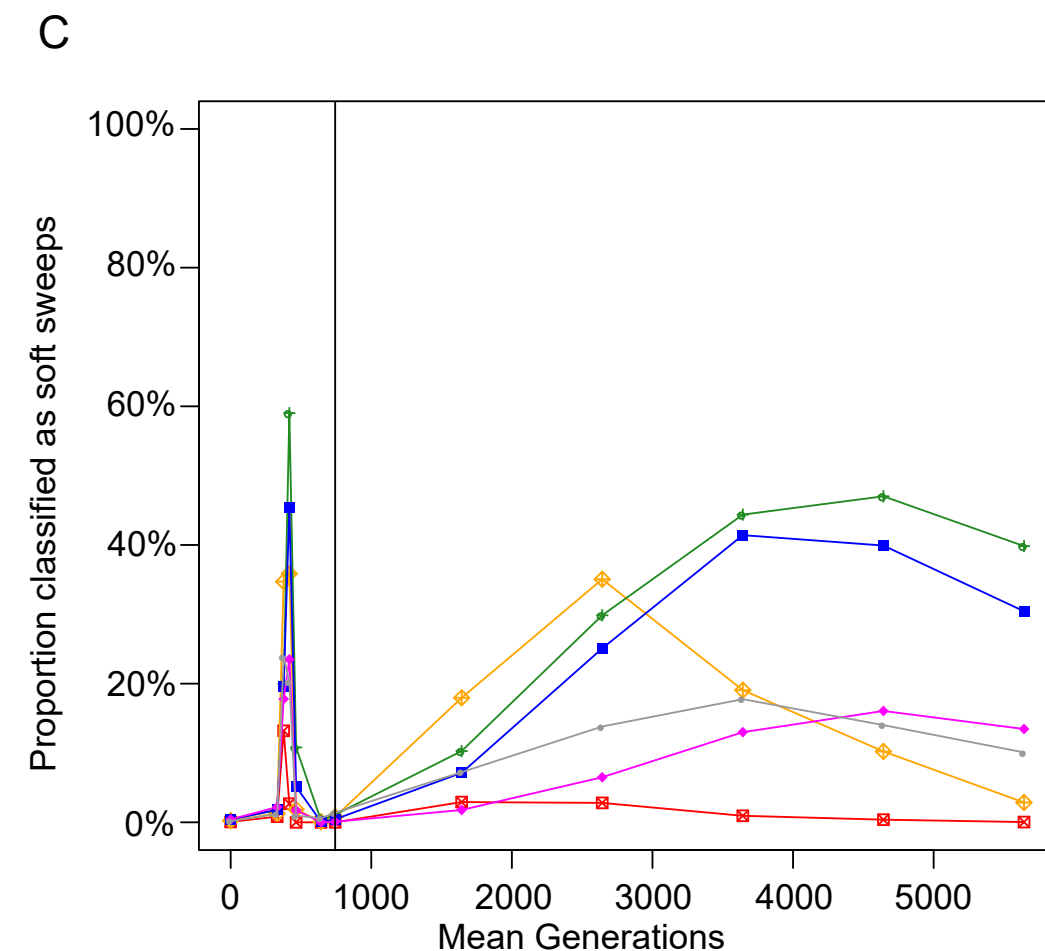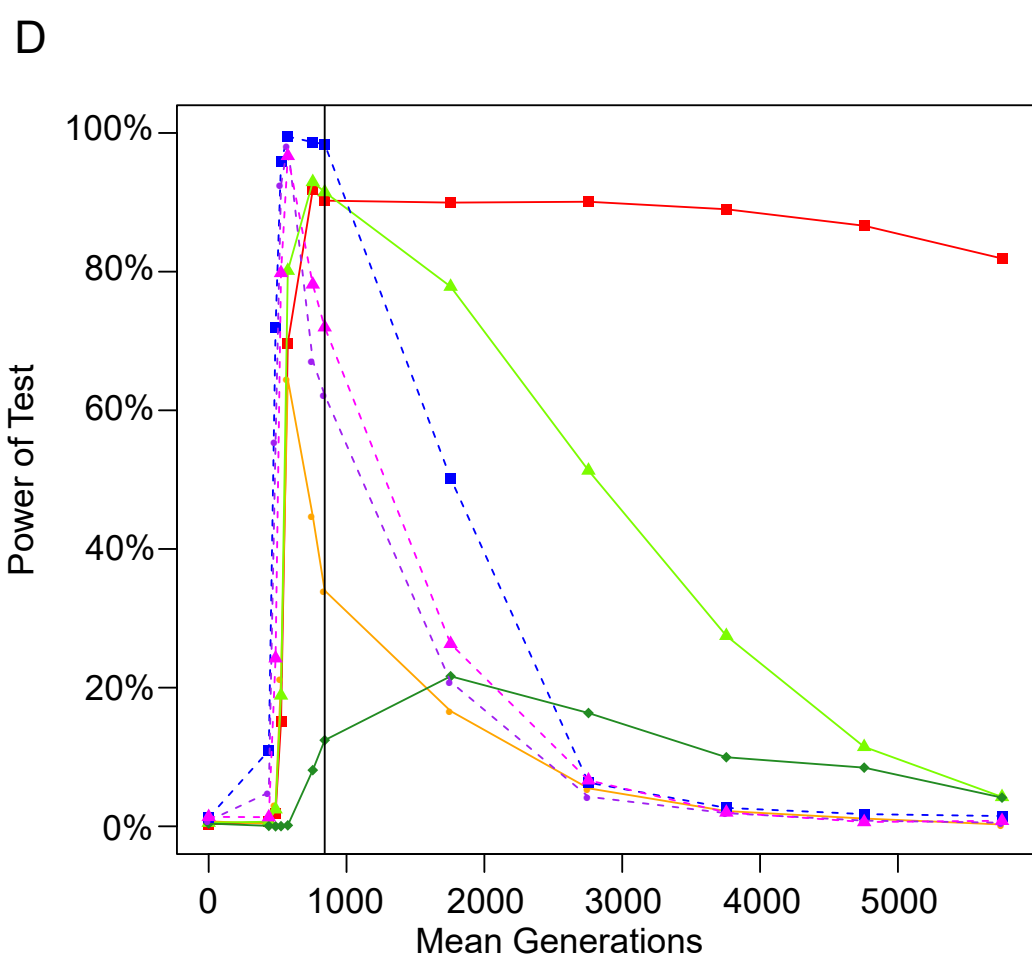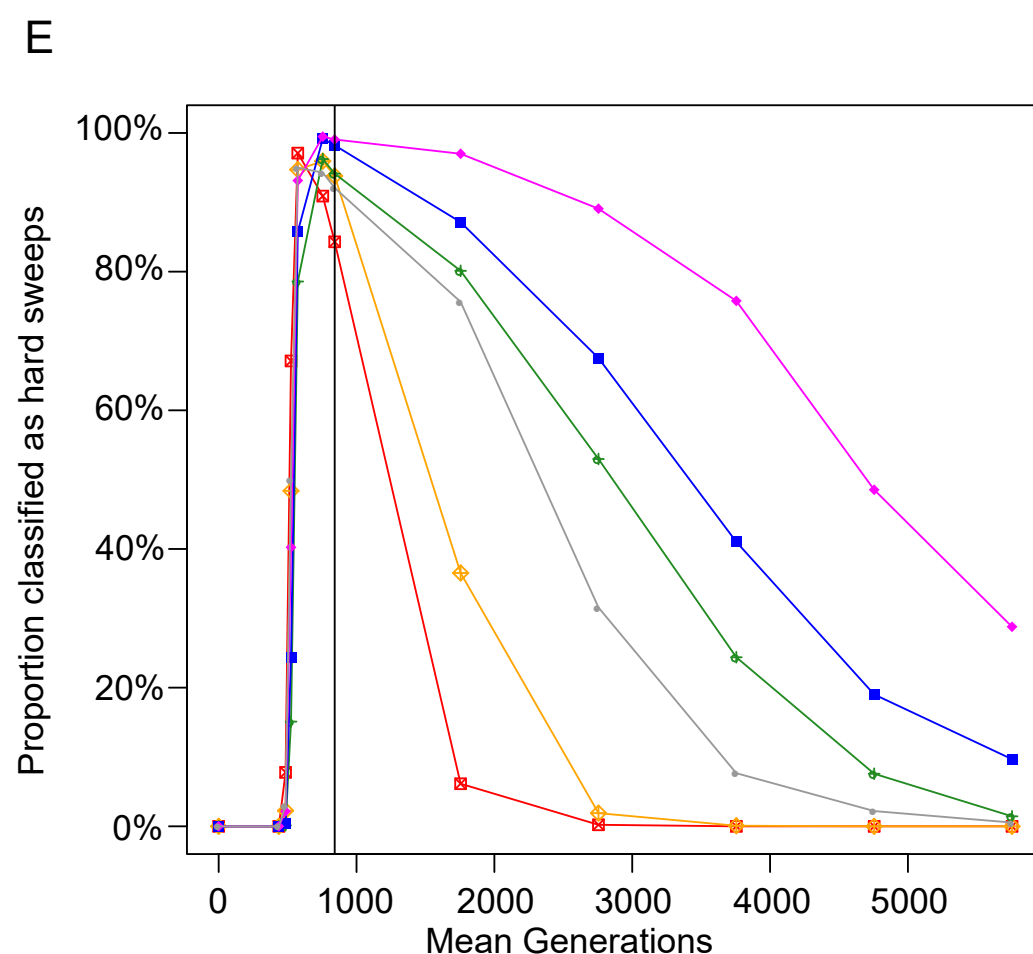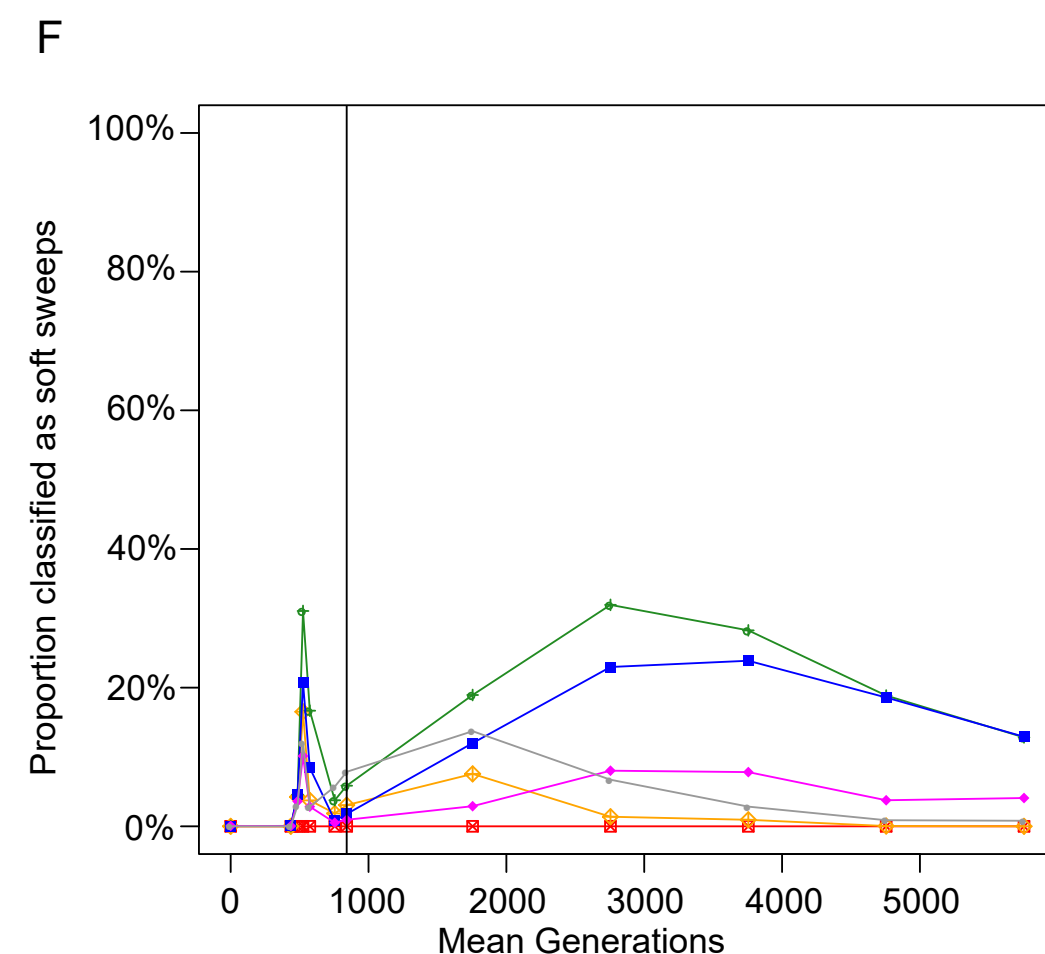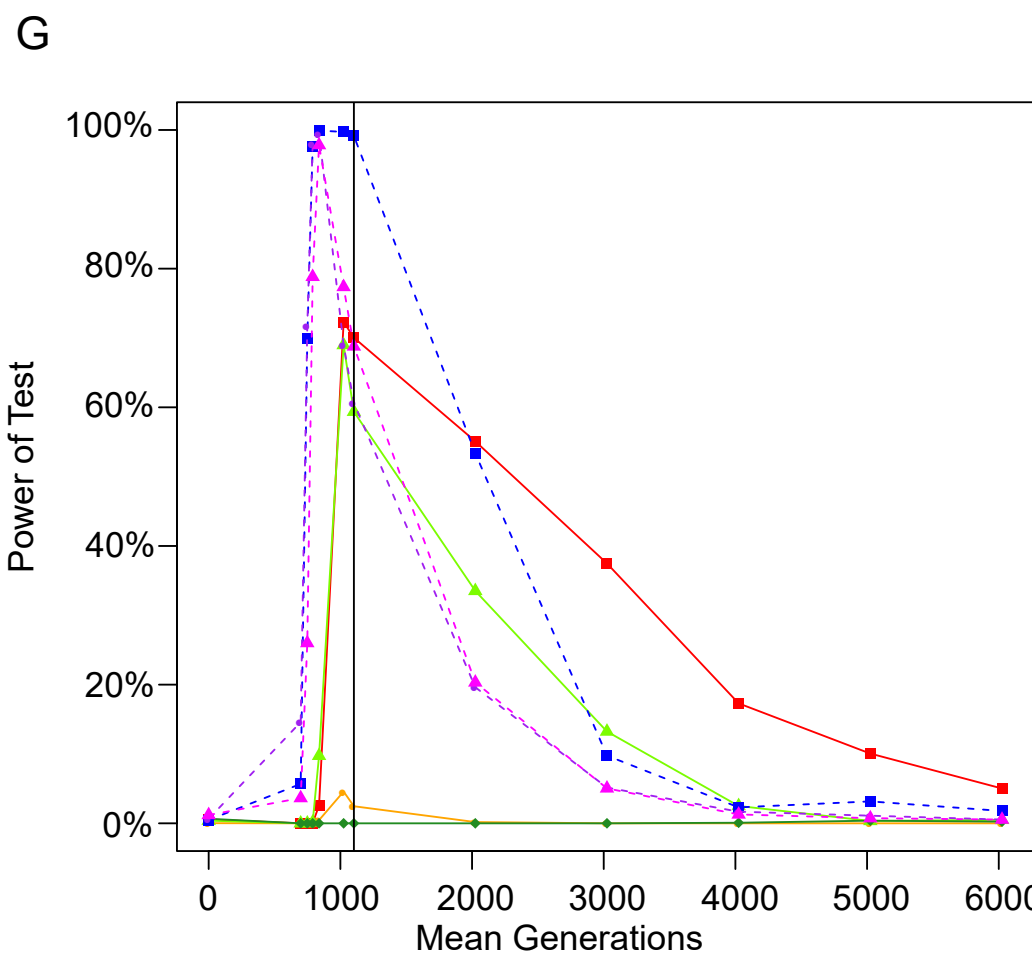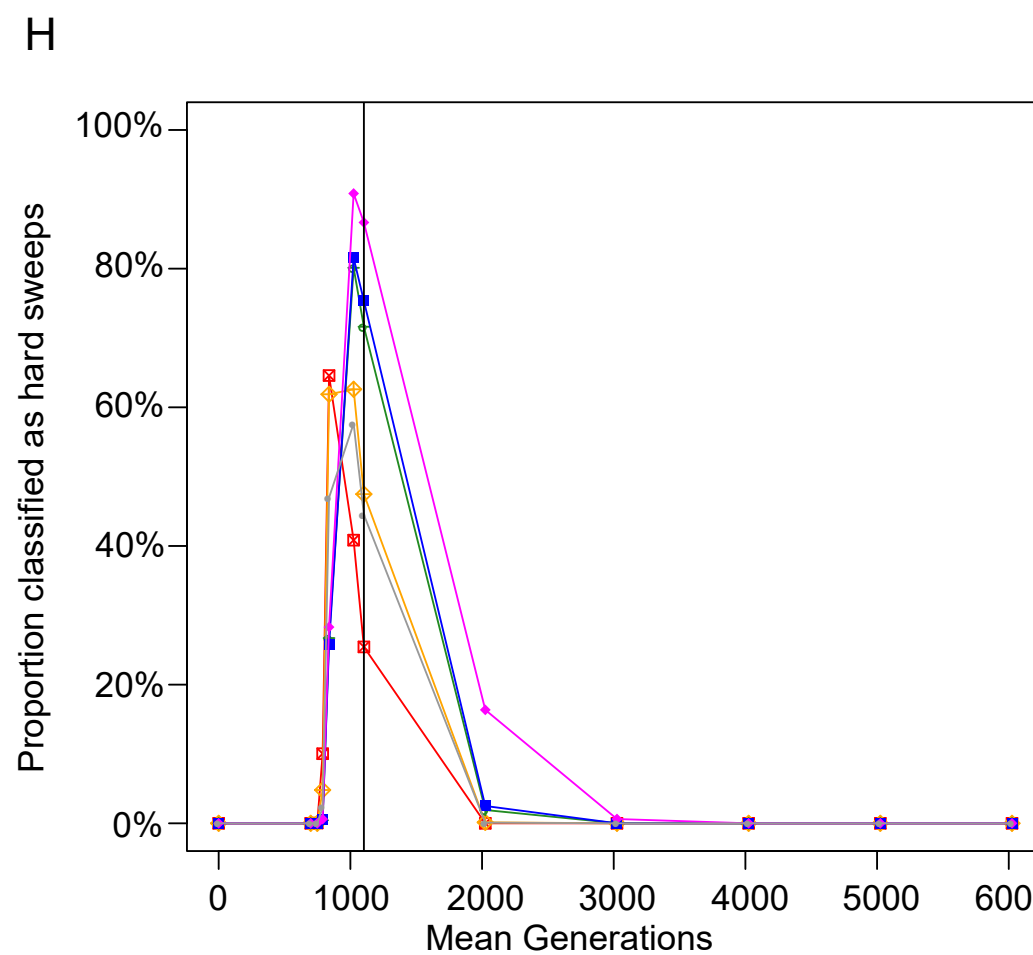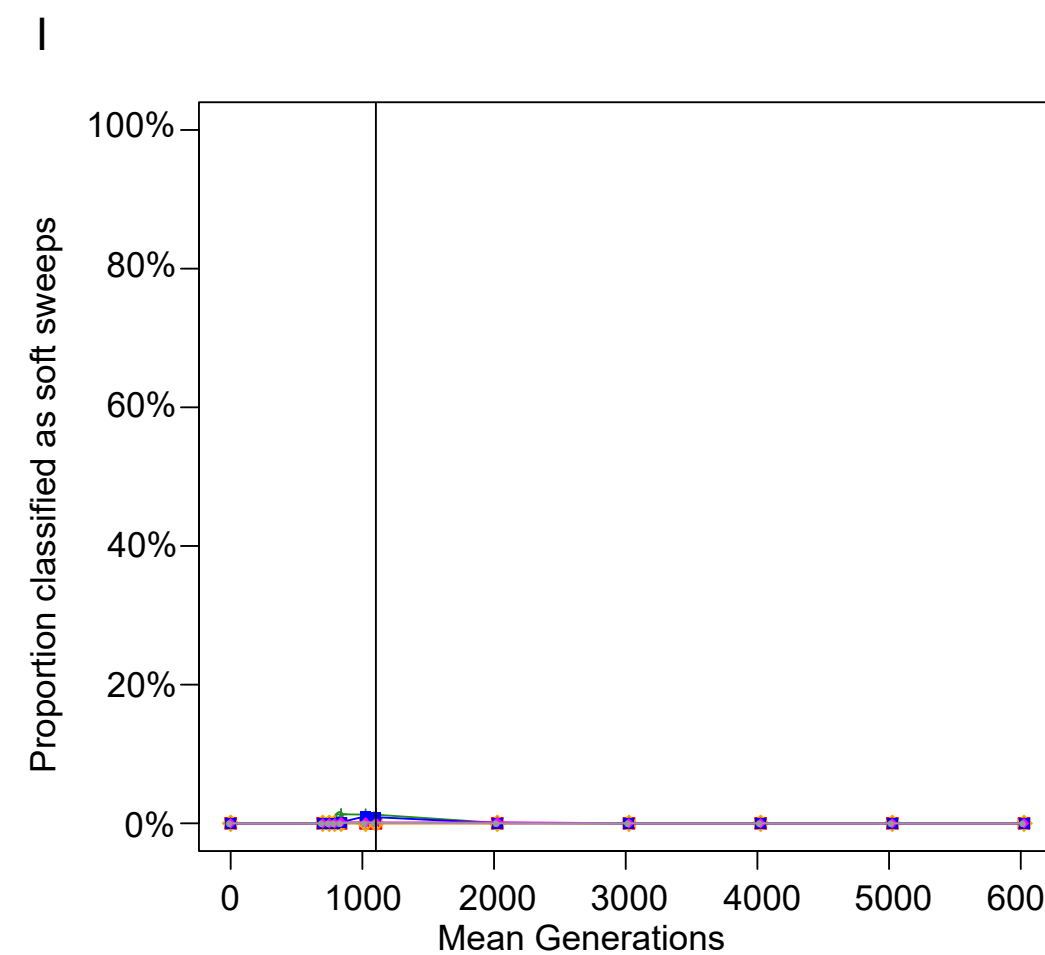

Supplement: S4 Fig — The proportion of samples detected as selective sweeps by various methods, under the scenarios: A-C. m20G, D-F. m2G, G-I. m0.2G, in d2 where the adaptive allele is imported to. The vertical line indicates time of 100% fixation. A,D,G. Power of seven summary statistics; dashed lines indicate haplotype-based methods. B,E,H. Proportion detected by six EvolBoosting predictors correctly as hard sweeps. C,F,I. Proportion detected by six EvolBoosting predictors incorrectly as soft sweeps. The general qualitative pattern is similar to d1. (PDF) [file pcbi.1007426.s007.pdf]
