# Supplementary material for: Adaptation in structured populations and fuzzy boundaries between hard and soft sweeps
Source: PLoS Comput Biol. 2019 Nov 11;15(11):e1007426. doi: 10.1371/journal.pcbi.1007426 (PMC6872172; doi:10.1371/journal.pcbi.1007426)
Supplement: S3 Fig — The proportion of samples detected as selective sweeps by various methods, under the scenarios: A-C. m0G, D-F. m20Ld1, G-I. m20Ld2, J-L. m0.2G. Only the time stages at or before global fixation are shown; the horizontal axis denotes adaptive allele frequency instead of generations. The vertical line indicates time of 100% fixation. A,D,G,J. Power of seven summary statistics; dashed lines indicate haplotype-based methods. B,E,H,K. Proportion detected by six EvolBoosting predictors correctly as hard sweeps. C,F,I,L. Proportion detected by six EvolBoosting predictors incorrectly as soft sweeps. The general qualitative pattern is similar to d1. (PDF) [file pcbi.1007426.s006.pdf]

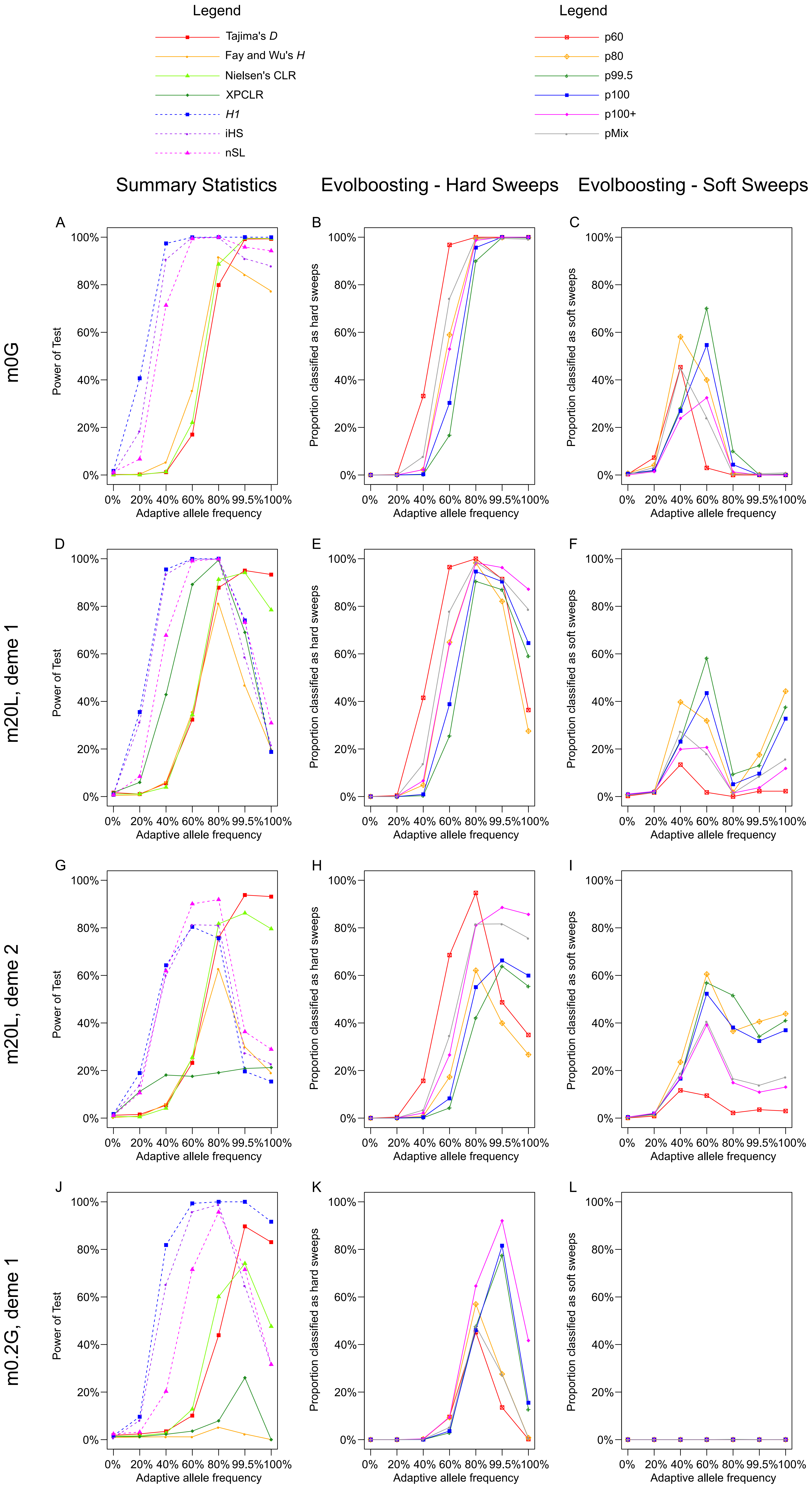

Legend
Tajima's D
Fay and Wu's H
Nielsen's CLR
XPCLR
H1
iHS
nSL
Legend
p60
p80
p99.5
p100
p100+
pMix
Summary Statistics
Evolboosting - Hard Sweeps
Evolboosting - Soft Sweeps
m0G
m20L, deme 1
m20L, deme 2
m0.2G, deme 1
A
B
C
D
E
F
G
H
I
J
K
L
Power of Test
Proportion classified as hard sweeps
Proportion classified as soft sweeps
Adaptive allele frequency
0%
20%
40%
60%
80%
99.5%
100%
